# Enzymatische C4‐Epimerisierung von UDP‐Glucuronsäure: präzise gesteuerte Rotation eines transienten 4‐Ketointermediats für eine invertierende Reaktion ohne Decarboxylierung

**DOI:** 10.1002/ange.202211937

**Published:** 2022-12-15

**Authors:** Annika J. E. Borg, Oriol Esquivias, Joan Coines, Carme Rovira, Bernd Nidetzky

**Affiliations:** ^1^ Institut für Biotechnologie und Bioprozesstechnik Technische Universität Graz Petersgasse 12/1 8010 Graz Österreich; ^2^ Austrian Center of Industrial Biotechnology (acib) Krenngasse 37 8010 Graz Österreich; ^3^ Department of Inorganic and Organic Chemistry (Section of Organic Chemistry) Institute of Computational and Theoretical Chemistry (IQTCUB) Martí i Franquès 1 08028 Barcelona Spanien; ^4^ Institució Catalana de Recerca i Estudis Avançats (ICREA) Passeig Lluís Companys, 23 08010 Barcelona Spanien; ^5^ Derzeitige Adresse: Nostrum Biodiscovery Av. De Josep Tarradellas, 8–10 08029 Barcelona Spanien

**Keywords:** Enzymkatalyse, Epimerase, Kohlenhydrate, QM/MM, UDP-Glucuronsäure

## Abstract

UDP‐Glucuronsäure(UDP‐GlcA)‐4‐Epimerase repräsentiert eine wichtige Fragestellung in der Enzymkatalyse: die Balance zwischen konformativer Flexibilität und genauer Positionierung. Das Enzym koordiniert die C4‐Oxidation des Substrats durch NAD^+^ mit der Rotation eines leicht decarboxylierbaren β‐Ketosäure‐Intermediats im aktiven Zentrum zur Ermöglichung der stereoinvertierenden Reduktion der Ketogruppe durch NADH. Wir zeigen hier die nur schwer erfassbare Rotationskoordinate des 4‐Ketointermediats. Distorsion des Zuckerrings in eine Boot‐Konformation erzeugt torsionale Mobilität in der Bindungstasche des Enzyms. Die Endpunkte der Rotation zeigen den 4‐Ketozucker in einer unverformten ^4^ *C*_1_‐Sesselkonformation. Die äquatorial positionierte Carboxylatgruppe ist ungünstig für die 4‐Ketozucker‐Decarboxylierung. Varianten der Epimerase zeigen Decarboxylierung, wenn sie die Bindung mit der Carboxylatgruppe im entgegengesetzten Rotationsisomer des Substrats entfernen. R185A/D‐Substitutionen wandeln die Epimerase in UDP‐Xylose‐Synthasen um, welche UDP‐GlcA in stereospezifischen, konfigurationserhaltenden Reaktionen decarboxylieren.

## Graphical abstract

Enzymatische C4‐Epimerisierung von UDP‐Glucuronsäure beinhaltet eine präzise gesteuerte Rotation eines transienten 4‐Ketohexuronsäure‐Intermediats, die durch Sessel‐zu‐Boot‐Distorsion des Zuckerrings erleichtert wird. Das Enzym erhält damit die notwendige stereoelektronische Kontrolle, um Decarboxylierung auch nur in Spuren zu vermeiden. Eine Verbindung zwischen konformativer Selektion und Epimerase‐ oder Decarboxylase‐Aktivität wird vorgeschlagen.

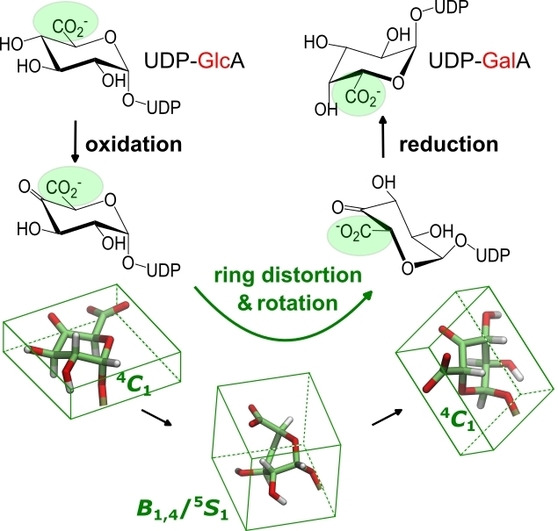

## Einleitung

Enzyme katalysieren chemische Reaktionen in unübertroffener Effizienz.^[1]^ In der modernen (d. h. dynamischen) Sichtweise der Enzymwirkung ist die konformative Auswahl (Sampling), welche durch die Proteinflexibilität ermöglicht wird, fundamental wichtig für Spezifität und Effizienz.^[2]^ Nicht nur ist das Sampling wesentlich für die Koordination des unmittelbar katalytischen Ereignisses mit anderen physikalischen Schritten der enzymatischen Reaktion, sondern es stellt auch einen Schlüssel zur dynamischen Population von Enzym‐Substrat‐Komplex‐Konformeren dar, welche für die Bindungsspaltung bzw. ‐knüpfung geeignete Elektrostatik und internukleare Distanzen aufweisen.[^2^, ^3^] UDP‐d‐Glucuronsäure(UDP‐GlcA)‐4‐Epimerase (UGAepi; EC 5.1.3.6) katalysiert die Stereoinversion am C4 der UDP‐GlcA, um d‐Galacturonsäure für die Biosynthese von Zellwandpolysacchariden zur Verfügung zu stellen.[^4^, ^5^, ^6^, ^7^, ^8^, ^9^] UGAepi repräsentiert in spezieller Weise eine allgemeine Fragestellung von fundamentaler Bedeutung in der Enzymkatalyse: Um die Reaktion effizient voranzutreiben, muss das Enzym eine feinabgestimmte Balance zwischen Proteinflexibilität und exakter Substratpositionierung erreichen.^[6]^

**Figure 1:**
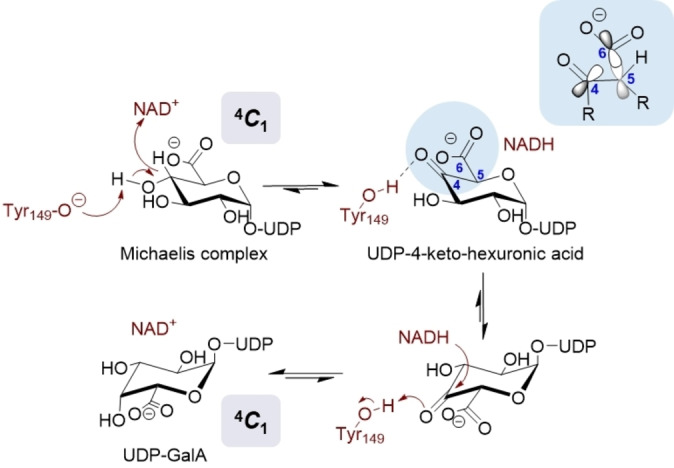
Vorgeschlagener Mechanismus der durch UGAepi katalysierten Umwandlung von UDP‐GlcA und UDP‐GalA unter Beteiligung von stereoelektronischer Kontrolle.^[6]^ Wechselwirkungen in der Bindetasche, welche die Carboxylatgruppe in einer äquatorialen Orientierung halten, wie in enzymgebundener UDP‐GlcA und UDP‐GalA^[5]^ beobachtet, würden zu einer die Decarboxylierung des 4‐Ketointermediats beungünstigenden Orbitalausrichtung führen.

Die UGAepi‐Reaktion besteht aus zwei katalytischen Schritten eines kanonischen Mechanismus einer Nukleotidzucker‐Epimerase,[^3h^, ^10^, ^11^, ^12^, ^13^, ^14^, ^15^] wie zum Beispiel UDP‐Galactose‐4‐Epimerase (Abbildung 1): ortsspezifische C4‐Oxidation des Substrats durch eng gebundenes NAD‐Coenzym; und nicht‐stereospezifische Reduktion eines transienten UDP‐4‐Ketohexuronsäure‐Intermediats durch Enzym‐NADH.[^12^, ^13^] Um die neuerliche Wasserstoffaddition von beiden Seiten der Carbonylgruppe zu ermöglichen, muss die 4‐Ketogruppe flexibel in der Bindungstasche des Enzyms untergebracht sein. Die generelle Ansicht ist hier, dass der 4‐Ketozucker zur Rotation im aktiven Zentrum des Enzyms in der Lage sein muss.[^4^, ^5^, ^6^, ^10^, ^11^, ^12^, ^13^, ^14^, ^15^] Wie jedoch das Enzym die torsionale Mobilität des gebundenen Intermediats ermöglicht, ist nicht verstanden und bleibt strukturell rätselhaft.

Die Rotation im enzymatischen Mechanismus erhält zusätzliche Bedeutung aus der Tatsache, dass UGAepi der Decarboxylierung einer metastabilen β‐Ketosäure‐Spezies, welche ihr 4‐Ketointermediat repräsentiert, entgegentreten muss.[^4^, ^6^] Es wurde vorgeschlagen, dass UGAepi Wechselwirkungen mit dem Substrat in der Bindungstasche (gezeigt in Kristallstrukturen des Enzyms in Komplex mit UDP‐GlcA und UDP‐GalA) ausnutzt, um die relative Orientierung der Carboxylat‐ und Ketogruppen des Intermediats so einzuschränken, dass eine Decarboxylierung stereoelektronisch beungünstigt ist.[^4^, ^5^, ^6^] Die Einbeziehung von konformativer Restriktion zum Zweck der stereoelektronischen Kontrolle erzeugt eine Schwierigkeit für das Enzym im Lichte der Notwendigkeit, eine freie Rotation des 4‐Ketointermediats zu ermöglichen. Hier berichten wir die Evidenz aus einer kombiniert computergestützten und experimentellen Analyse, welche den vollen Weg der Rotation des 4‐Ketointermediats nachzeichnet und die Verbindung der Rotation mit den katalytischen Schritten aufzeigt. Unsere Ergebnisse enthüllen die Notwendigkeit einer vom Enzym getriebenen Distorsion des Zuckerrings, um einerseits torsionale Mobilität zu induzieren und andererseits die Rotation der 4‐Ketohexuronsäure‐Gruppe in der eingeengten Bindungstasche der UGAepi zu steuern. Eine Rolle für konformatives Sampling verbunden mit chemoselektiver Katalyse der Epimerase wird daher vorgeschlagen. Eine wichtige Entdeckung der Arbeit ist außerdem, dass Varianten der UGAepi, hergestellt mit dem Ziel, einen wesentlichen Defekt im konformativen Sampling des Enzyms zu erzeugen, sich wie primitive Decarboxylasen verhalten, welche die langsame Freisetzung von UDP‐Xylose aus UDP‐GlcA in völlig stereospezifischen Reaktionen vorantreiben. Insgesamt betont unsere Studie am Beispiel der UGAepi die Bedeutung von koordinierten Änderungen in der Proteinkonformation (gekoppelte Bewegungen; coupled motions) für die Effizienz von mehrstufiger Enzymkatalyse.[^2a^, ^2f^, ^3^, ^6^]

## Ergebnisse und Diskussion

**Figure 2:**
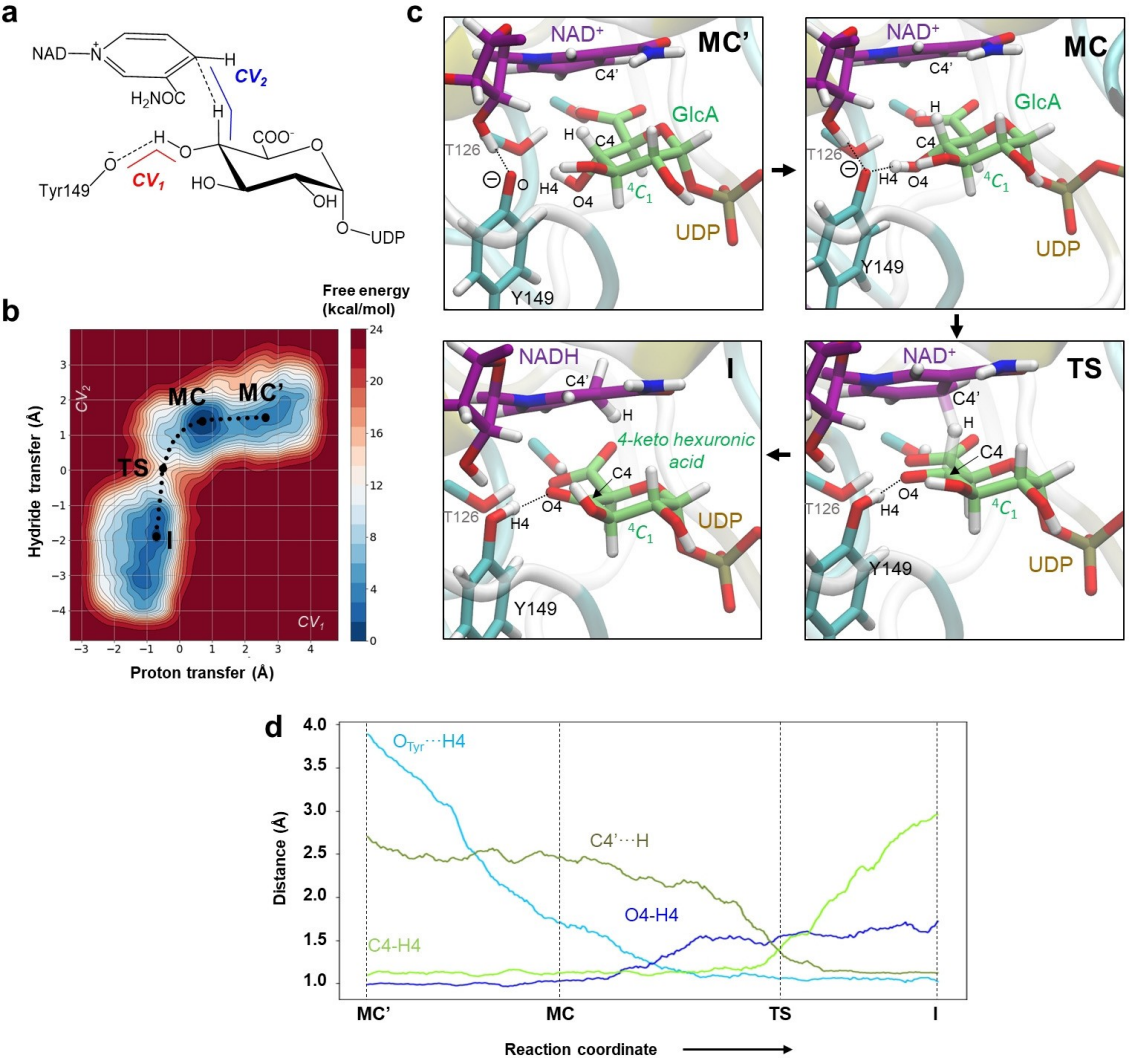
QM/MM‐Metadynamics‐Simulationen der Oxidation von UDP‐GlcA katalysiert durch BcUGAepi. a) Die verwendeten kollektiven Variablen. b) Freie‐Energie‐Landschaft (FEL) erhalten aus der Simulation (Isolinien bei 1 kcal mol^−1^). c) Repräsentative Strukturen von stationären Zuständen entlang der Reaktionskoordinate. d) Evolution der hauptsächlichen katalytischen Distanzen entlang eines minimalen Wegs der freien Energie (Reaktionskoordinate).

In einem ersten Schritt unserer Untersuchung analysierten wir die katalytische Konversion von UDP‐GlcA in das 4‐Ketointermediat mittels MD‐ und QM/MM‐Metadynamics‐Methoden (Hintergrundinformationen methods). MD‐Simulationen (600 ns) ausgehend von der Struktur des UDP‐GlcA‐Komplexes von BcUGAepi (Enzym von *Bacillus cereus*; PDB 6ZLD^[5]^) zeigten die Pyranosylgruppe in einer durchwegs entspannten ^4^ *C*_1_‐Konformation. Der C4‐Wasserstoff des Zuckers bleibt in enger Nachbarschaft (2.6 Å Abstand im Mittel) zum Nikotinamid‐C4′‐Atom (Abbildung S1). Gleichzeitig ist die 4‐OH häufig in eine Wasserstoffbrückeninteraktion mit dem Phenolat des Y149 eingebunden (H4⋅⋅⋅O_Tyr_=1.9 Å, Abbildung S1). Y149 ist hochkonserviert, und seine vorgeschlagene Funktion ist die einer allgemeinen Base für die Oxidation.^[4]^ Da die beteiligten Gruppen eine plausible Ausrichtung für die C−H‐Bindungsspaltung unter protonischer Mitwirkung aufweisen, erscheinen die beobachteten Konformationen als Bona‐fide‐Repräsentanten des BcUGAepi‐Michaelis‐Komplexes (Abbildung S1). Eine Konformation, entsprechend einem Schnappschuss der dynamisch äquilibrierten Proteinstruktur, wurde für eine detaillierte Untersuchung des katalytischen Prozesses mittels QM/MM‐Metadynamics ausgesucht. Eine große QM‐Region (101 QM‐Atome; 1 082 223 MM‐Atome) wurde in Betracht gezogen, einschließlich der GlcA‐Einheit, der Phosphate des UDP, Teile des NAD^+^ und der Seitenketten von Y149 und T126. Zwei kollektive Variablen (CV) wurden definiert, um die Protonen (CV_1_) und die Hydrid‐Abstraktion (CV_2_) der gesamten Oxidation zu beschreiben (Abbildung 2a).

Ergebnisse in Abbildung 2b,c zeigen, dass sich die Reaktion effektiv vom vorgeschlagenen Michaelis‐Komplex (erwähnt als **MC**) hin zum UDP‐4‐Ketohexuronsäure/NADH‐Komplex (erwähnt als **I**, für Intermediat) entwickelt. Die zwei Minima der freien Energie, **MC** und **I** zugeordnet, sind praktisch isoenergetisch (Unterschied <1 kcal mol^−1^) und durch einen eindeutigen Übergangszustand getrennt (**TS**), welcher auf eine konzertierte Reaktion hindeutet. Die freie Energie des katalytischen Schritts (**MC**→**TS**) ist 13.1 kcal mol^−1^. Unter Verwendung der Transition State Theory entspricht diese Energiebarriere einer Geschwindigkeitskonstante für die unmittelbare Katalyse, welche ≈10^3^‐fach schneller ist als der experimentelle *k*_cat_ (≈1 s^−1^; bei 300 K). Der beobachtete *k*_cat_ ist jedoch nicht allein durch den katalytischen Schritt limitiert. Kinetische Isotopeneffekte implizieren eine präkatalytische Umlagerung des Enzym‐Substrat‐Komplexes in der partiellen Geschwindigkeitslimitierung des *k*_cat_.^[4]^ Kristallstrukturen der UGAepi geben einen zusätzlichen Hinweis zur Unterstützung der “kinetischen Komplexität” des *k*_cat_, indem sie eine Änderung der Proteinkonformation (Bewegung einer Proteinschleife, die zum Abschließen des aktiven Zentrums führt) in Assoziation mit der Bindung von UDP‐GlcA aufzeigen.^[5]^ Es ist wichtig zu beachten, dass unsere QM/MM‐Metadynamics‐Berechnungen von einer geschlossenen (closed‐loop) Struktur des Enzym/UDP‐GlcA‐Komplexes ausgehen und dass daher die konformative Änderung nicht modelliert wurde.

Nichtsdestotrotz zeigen die Berechnungen, dass die Bildung von **MC** den Übergang von einem sekundären Minimum (**MC**′), entsprechend einem Enzymkonformer, in dem das Y149‐Phenolat mit dem Ribosyl‐2‐OH des NAD^+^ wasserstoffverbrückt ist (Abbildung 2c), beinhaltet. Obwohl die **MC′**‐Konformere nicht produktiv für die Oxidation sind, hat ihr Auftreten mechanistische Bedeutung in zweierlei Hinsicht. Erstens, so wird angenommen, ist die flexible Orientierung von Y149 wie in **MC** und **MC′** wichtig für ein Protonenrelais in der Katalyse jener Superfamilie an Enzymen (short‐chain dehydrogenases/reductases; SDRs), zu denen auch UGAepi gehört.^[16]^ Zweitens: Neben der Energiebarriere (**MC**→**TS**) enthält der experimentell beobachtete *k*_cat_ auch den Effekt der nicht‐produktiven Enzymzustände. In anderen Worten, der computerunterstützt vorhergesagte *k*_cat_ wird reduziert einerseits durch den Anteil an nicht‐produktiven Konformeren wie **MC′** im gesamten Enzym und andererseits durch den Beitrag des Umlagerungsschrittes im Zuge der Substratbindung. Wir halten fest, dass Wasserstoff‐Deuterium‐Austausch eine interessante Technik sein kann, um in zukünftigen Studien die konformativen Änderungen assoziiert mit dem unmittelbaren chemischen Ereignis der Katalyse von BcUGAepi zu untersuchen.^[2c]^

Der Weg minimaler freier Energie für die katalytische Oxidation (Abbildung 2b,d) zeigt eine asynchrone Reaktion, deren Hydridtransfer weit hinter dem Protonentransfer hinterherhinkt. Der **MC** weist eine ausgeprägt kurze Wasserstoffbrücke (Y149−O⋅⋅⋅H4=1.7 Å) auf, welche den Protonentransfer initiiert (Tabelle S1). Im Zuge des Fortschritts zum **TS** ist das Proton vollständig transferiert, während der Hydridtransfer etwa auf halbem Weg zwischen den Kohlenstoffen des Substrats und des NAD^+^ (Abbildung 2d) ist. Wie aus der Form der freien Energielandschaft vorgeschlagen (Abbildung 2b), ist es hauptsächlich der Hydridtransfer, welcher Aktivierungsenergie benötigt, um voranzuschreiten. Ausgenommen die erhöhte Ringplanarität, verursacht durch die 4‐Ketogruppe und gemessen mittels der so genannten “radial puckering”‐Koordinate (Abbildung S2), bleibt die Hexuronsäure‐Gruppe durchwegs in der ^4^ *C*_1_‐Konformation.

**Figure 3:**
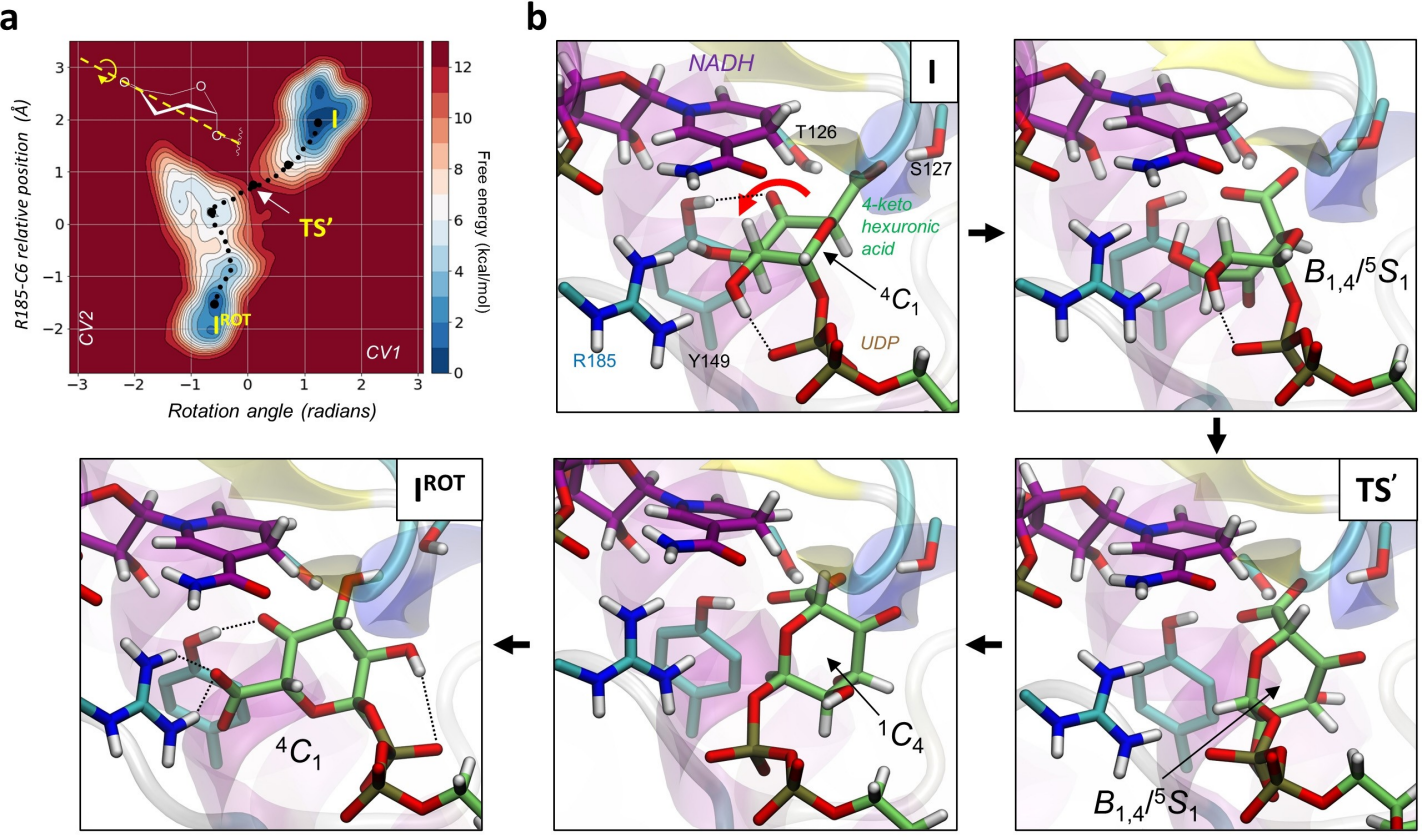
Rotationskoordinate für die 4‐Ketohexuronsäure im aktiven Zentrum der BcUGAepi, erhalten aus Metadynamics‐Simulationen. a) FEL aus einer repräsentativen Metadynamics‐Simulation der reversiblen Rotation. Beachte Tabelle S3 für die konsistente Reversibilität der Rotation zwischen **I** und **I^ROT^**. b) Repräsentative Strukturen von stationären und relevanten Zuständen entlang der Reaktionskoordinate. Eine volle Darstellung der strukturellen Änderungen des Rotationswegs ist im Supporting Movie gezeigt.

Ausgehend von dem entstehenden **I**‐Konformer modellierten wir die Rotation des 4‐Ketointermediats. Da keine kovalente Bindung im Verlauf des Prozesses gebrochen/gebildet wird, sind Kraftfeld‐basierte Rechenmethoden (klassische MD mit Metadynamik) geeignet für die Analyse. Aufgrund des Fehlens eines Kraftfelds zur Beschreibung der 4‐Ketohexuronsäure‐Gruppe entwickelten wir eines für die spezifische Aufgabe (Hintergrundinformationen methods). Die Struktur des BcUGAepi‐Komplexes mit UDP‐GalA (PDB 6ZLL^[5]^) diente als Referenz für die Bewertung des Konformers im Endpunkt der Rotation. Vorläufige Evidenz zeigte, dass eine einzelne CV (der Drehwinkel um die longitudinale Achse des Zuckerrings) nicht imstande war, Enzymkonformere zu erhalten, die eine volle Rotation des Pyranosylrings aufwiesen, so dass die Carboxylatgruppe an der entgegengesetzten Seite positioniert wäre, um mit R185 zu interagieren. Hinzunahme einer zweiten CV, mit dem Ziel, das Carboxylat zur Seitenkette des R185 zu ziehen, war erfolgreich, die Rotationsbewegung vollständig voranzutreiben (Abbildung S3). Die zwei Endpunkte der reversiblen Rotation (**I**, **I^ROT^**) entsprechen isoenergetischen Minima der freien Energie in der konformativen Landschaft. Sie sind durch eine Barriere an freier Energie (**TS′**) von 10.8 kcal mol^−1^ verbunden (Abbildung 3a). Basierend auf Unterschieden in der Höhe der Energiebarriere (+2.3 kcal mol^−1^) kann erwartet werden, dass die Rotation ≈60‐fach schneller ist als die Oxidation. Dieser Befund ist konsistent mit früheren biochemischen Evidenzen,^[4]^ die zeigen, dass die reduzierte Enzymform BcUGAepi‐NADH unterhalb der Nachweisgrenze während der Epimerisierung von UDP‐GlcA im Fließgleichgewicht liegt, was wiederum impliziert, dass sowohl die Rotation wie auch die Reduktion unter Inversion der Konfiguration schnell im Vergleich zur Oxidation ablaufen. Detaillierte Analyse der Rotationskoordinate (Abbildung 3b) identifiziert eine komplexe molekulare Bewegung (coupled motion), die substanziell von der Rotation eines festen Körpers (rigid body) abweicht. Diese Bewegung beinhaltet eine koordinierte Serie von Konformationen der 4‐Ketohexuronsäure‐Gruppe innerhalb der Bindetasche des Enzyms (beachte den Supporting Movie). Gelenkt durch eine Wasserstoffbrücke von S127 und später Y149 zum Carboxylat des Zuckers (Abbildung 3b) verzerrt sich der Pyranosylring von einer initialen ^4^ *C*_1_‐Konformation in eine hochenergetische Konformation, die zwischen den *B*_1,4_‐ und ^5^ *S*_1_‐Konformationen liegt. Die konformative Umlagerung orientiert die Carboxylatgruppe axial mit der Konsequenz für die Rotation, dass das zugängliche Volumen des Zuckers reduziert wird und die sterischen Wechselwirkungen mit dem Dihydronikotinamidring minimiert werden.

Die katalytisch relevante Wasserstoffbrücke von Y149 zur 4‐Ketogruppe (ein charakteristisches Merkmal von **I** wie auch **I^ROT^**) wird im Zuge der Rotation gebrochen. Trotz der extensiven Änderungen in der Faltung (pucker) des an der Rotation beteiligten Zuckerrings erhält die 4‐Ketohexuronsäure die stabilisierenden Wechselwirkungen ihrer 2‐OH mit dem β‐Phosphat des UDP (Abbildung S4). Der **TS′**‐Zustand zeigt eine verzerrte *B*_1,4_/^5^ *S*_1_‐Konformation. Die Simulationsergebnisse geben Hinweise auf einen ≥10 kcal mol^−1^ energetischen Vorteil einer Rotation über den Weg von **TS′** im Vergleich zu einer einfachen Rotation als fester Körper (dargestellt durch ein “Einfrieren” der Distorsion des Zuckerrings, beachte Abbildung S5). Nachdem der **TS′** überschritten ist, bewegt sich die Pyranosylgruppe auf eine invertierte Sesselkonformation (^1^ *C*_4_) zu, wobei das Carboxylat in einer axialen Orientierung und wasserstoffverbrückt mit Y149 gehalten wird (Abbildung 3b). Sobald jedoch das Umklappen (flip) des Rings komplett erfolgt ist, kann sich der Zucker zurück in eine ^4^ *C*_1_‐Sesselkonformation entspannen, welche das Carboxylat wiederum äquatorial positioniert und es ihm so ermöglicht, eine starke Interaktion mit R185 einzugehen (Abbildung 3b, **I^ROT^**). In beiden Konformeren **I** und **I^ROT^** sind daher die stereoelektronischen Voraussetzungen gegeben, dass eine Decarboxylierung der β‐Ketosäure‐Spezies vermieden werden kann. Das äquatoriale Carboxylat bringt die Cα‐CO_2_^−^‐Bindung in etwa in eine Ebene mit der C=O‐Bindung des Ketons. Orbitalüberlappung ist jedoch optimal für die Decarboxylierung, wenn die beiden Bindungen grob orthogonal zueinander sind, was für ein axial orientiertes Carboxylat der Fall wäre.^[6]^ Um den Verlust an stereoelektronischer Kontrolle in jenen Konformeren auszugleichen, die eine Distorsion der Zuckerringfaltung aufweisen, mag das Enzym auf eine unterscheidende Wasserstoffbrückenbindung zurückgreifen, die ein gleichsames “Festhalten” des axialen Carboxylats ermöglicht. Wenn das Y149 von der 4‐Ketogruppe weg orientiert und auf das Carboxylat ausgerichtet ist, dann ist ein Elektronenfluss in das C5 und das O4 abgeschwächt und die Bereitschaft zur Decarboxylierung kann dadurch effektiv unterdrückt sein. Das **I^ROT^**‐Konformer involviert analoge Wechselwirkungen von Relevanz für die Katalyse wie **I**, und es erscheint völlig bereit für die Reduktion der 4‐Ketogruppe, um damit die C4‐Epimerisierung zu komplettieren, ohne dass dabei Decarboxylierung auch nur in Spuren auftreten würde. Der finale Reduktionsschritt wurde daher nicht mehr analysiert.

Basierend auf der Evidenz der Computerberechnungen wandten wir Mutagenese an (Hintergrundinformationen methods), um Wechselwirkungen der Bindetasche des Enzyms mit dem Carboxylat des 4‐Ketointermediats in **I** und **I^ROT^** zu untersuchen. In **I** (Abbildung S6) ist das Carboxylat durch T126, S127, S128 und T178 (NH der Hauptkette) koordiniert. In **I^ROT^** (Abbildung S6) ist es durch R185 koordiniert. Sequenzvergleich (Abbildung S7) zeigt, dass S128, T178 und R185 einzigartig für die UGAepi‐Unterklasse der SDRs sind. Aminosäurereste wurden individuell substituiert, um stabilisierende Interaktionen zu entfernen oder destabilisierende Interaktionen einzuführen. Im Falle von R185 führte das zu einer Serie von Varianten, von denen angenommen wird, dass sie eine abgestufte Änderung von Stabilisierung (Wildtyp>R185K>R185H) zu Destabilisierung (R185D>R185A) darstellen. Gereinigte Enzyme (Abbildungen S8 und S9), von denen bestätigt wurde, dass sie NAD^+^ in das gefaltete Protein eingebaut haben, wurden in Reaktionen mit UDP‐GlcA (1.0 mM) getestet, wobei HPLC (Abbildungen S10–S13) und NMR für die Analyse eingesetzt wurden. Tabelle 1 fasst die Resultate zusammen. Die gesamten Zeitverläufe sind in Abbildungen S14–S22 gezeigt.

**Table 1:** Aktivitäten und Produktverhältnisse für Varianten der BcUGAepi in Reaktionen mit UDP‐GlcA (**1**) und UDP‐GalA (**2**). Die Aktivitäten wurden aus dem linearen Teil des Zeitverlaufs bestimmt, wobei der initiale Burst in einigen Reaktionen ausgeklammert wurde (Abbildungen S14–S22 und S28–S30). Die Steigung aus der linearen Regression (mM min^−1^) wurde mit der Enzymkonzentration (mg mL^−1^) dividiert, um die Initialrate in μmol(min mg)^−1^ zu erhalten. Diese Rate entspricht U mg^−1^.

Enzym	Substrat	Aktivität [mU mg^−1^]	UDP‐GlcA (**1**, %)	UDP‐GalA (**2**, %)	UDP‐Xylose (**3**, %)	UDP‐4‐Ketopentose (**4**, %)	Burst^[e]^
Wildtyp	**1**	500	33	67	0	0	–
T126A	**1**	0.08	33^[a]^	67^[a]^	0^[a]^	0^[a]^	–
S127A	**1**	24.3	33	67	0	0	–
S128A	**1**	11.8	33	67	0	0	–
S128E	**1**	0.2	85.4^[b]^	12.1^[b]^	1.7^[b]^	0.8^[b]^	–
T178A	**1**	0.09	33^[a]^	67^[a]^	0^[a]^	0^[a]^	–
R185A	**1**	0.05	90.7^[c]^	0^[c]^	0^[c]^	9.3^[c]^	0.10
R185D	**1**	0.05 (≈2.6)	92.5^[c]^	0^[c]^	5.1^[c]^ (≈95)	2.4^[c]^ (≈5)	0.22
R185H	**1**	0.05 (≈5)	81.3^c]^	8.1^[c]^	9.0^[c]^ (≈95)	1.6^[c]^ (≈5)	2.43
R185K	**1**	0.3 (≈66)	51.1^[c]^	21.8^[c]^	26.8^[c]^ (≈98)	0.3^[c]^ (≈2)	–
Wildtyp	**2**	500	33	67	0	0	–
T126A	**2**	0.09	24.8	74.8	0	0.4	–
S127A	**2**	218	29.1	70.5	0.4^[d]^	0	–
R185H	**2**	0.1	18.3	70.7	5.0^[d]^	6.0	–

Wenn nicht anders angemerkt, sind die berichteten Verhältnisse nach 24 h Reaktionszeit. Für die R185H‐, R185D‐ und R185K‐Enzyme sind die Aktivität sowie die Zusammensetzung des initialen Produktbursts (nach 1 min) in Klammern angegeben. Jede Reaktion wurde zumindest in Duplikaten durchgeführt, wobei die Genauigkeit der Daten bei ±5 % iegt. [a] Produktverhältnisse nach 48 h Reaktion. [b] Produktverhältnisse nach 2 h Reaktion. [c] Produktverhältnisse nach einer Reaktion über 2 h 30 min. [d] Das Produkt ist UDP‐Pentose (UDP‐Xylose und/oder UDP‐l‐Arabinose). [e] Gesamte Molarität des Produktbursts nach 1 min (μM), dividiert durch die Enzymmolarität (μM). Der Burst bezieht sich hier auf eine spezifische Zeit der Probenahme, um die schnelle initiale Produktfreisetzung zu erfassen. Diese Probenahme mag für die Analyse des ersten Enzymumsatzes (turnover) nicht ausreichend gewesen sein (beachte den Eintrag für die R185H‐Variante). Anmerkung: Spezifische Aktivitäten wurden hier anstelle eines kompletten Sets von kinetischen Parametern (*k*_cat_, *K*_m_) bestimmt. Dieser Ansatz wurde im Hinblick auf den Fokus der Studie auf die Analyse der Produktbildung verfolgt.

Die spezifische Aktivität war in allen Enzymvarianten ≥10^3^‐fach reduziert, ausgenommen S127A und S128A, die ≈25‐ und ≈50‐fach weniger aktiv waren als der Wildtyp. Die gebildeten Produkte waren abhängig davon, ob die Mutation auf die Interaktionen im **I**‐ oder **I^ROT^**‐Konformer abgezielt hatte. Auf das **I**‐Konformer abzielende Varianten setzten die UDP‐GlcA sauber in UDP‐GalA um, außer die S128E‐Variante, welche Spuren von Decarboxylierung zeigte (Abbildung S10). Markanterweise ergaben die das **I^ROT^**‐Konformer beeinträchtigenden R185‐Varianten prädominant Decarboxylierung in ungefähr der Hälfte (R185K, R185H) oder der gesamten UDP‐GlcA (R185A, R185D), die in der Reaktion umgesetzt wurde (Tabelle 1; Abbildung S10). Der Anteil des Substrats, welcher der Decarboxylierung in den R185K‐ und R185H‐Reaktionen entging, wurde unter C4‐Stereoinversion in UDP‐GalA umgesetzt. Das decarboxylierte Produkt war aus UDP‐4‐Ketopentose und UDP‐Xylose zusammengesetzt, wobei deren relative Mengen unter den verschiedenen Enzymen variierten (Tabelle 1). R185A produzierte nur UDP‐4‐Ketopentose, während R185K UDP‐Xylose mit Spuren an UDP‐4‐Ketopentose produzierte. R185H und R185D produzierten Mischungen aus UDP‐Xylose/UDP‐4‐Ketopentose in einem Verhältnis von jeweils 5,6 : 1 und 2,1 : 1 NMR‐Daten zeigten die Identität von UDP‐Xylose und konnten UDP‐l‐Arabinose ausschließen (Abbildung S23). Reduktion der UDP‐4‐Ketopentose war daher stereospezifisch und erhielt die Konfiguration am C4 des UDP‐GlcA‐Substrats.

Die Reaktion von bestimmten R185‐Varianten (H, K, D) zeigte eine ungewöhnliche Kinetik: Einer schnellen initialen Freisetzung (referenziert als “Burst”) von decarboxyliertem Produkt (UDP‐Xylose, UDP‐4‐Ketopentose) war eine ≈10^2^‐fach langsamere Bildung der/s Produkte/s mit der Zeit nachgeschaltet (Tabelle 1). Bedeutend ist, dass die Burst‐Phase ausschließlich Decarboxylierung beinhaltete, während die Reaktion im Fließgleichgewicht die epimerisierte UDP‐GalA zusätzlich lieferte, und zwar in einem relativen Anteil am gesamten Produkt, der stark von dem verwendeten Enzym abhängig war. Aus einer Serie von kinetischen Experimenten mit BcUGAepi (Hintergrundinformationen methods; Abbildungen S24, S25) identifizierten wir enge Bindung/langsame Freisetzung von UDP‐Xylose als den wahrscheinlichen Grund für das abrupte Nachlassen der Reaktion bei hohen Enzymkonzentrationen. Der Burst konnte eliminiert werden, wenn UDP‐Xylose am Start der Reaktion zugegeben wurde. Ein plausibler kinetischer Mechanismus für die gemischte Decarboxylierung‐Epimerisierung der R185‐Varianten ist in Schema 1 gezeigt.

**Scheme 1:**
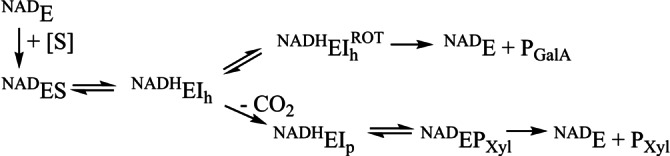
Vorgeschlagener kinetischer Mechanismus für die Decarboxylierungs‐Epimerisierungs‐Reaktion der R185‐Varianten. E=Enzym; S=Substrat; h=Hexose; p=Pentose.

Die angesammelte Evidenz aus der Studie der Enzymvarianten unterstützt die Idee, dass Decarboxylierung in jenem Ausmaß auftritt, in dem die ortsgerichtete Substitution das **I^ROT^**‐Konformer destabilisiert. In der Suche nach zusätzlicher Unterstützung durch eine unabhängige Methodik analysierten wir mittels MD‐Simulationen die **I^ROT^**‐Konformere in allen hier evaluierten R185‐Varianten. Die Resultate erklären die charakteristischen Verluste an Aktivität und Selektivität (Epimerase im Vergleich zu Decarboxylase) in den Enzymvarianten als entstanden aus jeweils einer niedrigeren Präzision in der Positionierung für die Katalyse und einem Fehlen an konformativer Beschränkung für eine enge stereoelektronische Kontrolle. Die für die unmittelbare Katalyse relevanten Interatomdistanzen werden in den simulierten **I^ROT^**‐Konformeren als verlängert vorgefunden, wenn im Besonderen die Varianten mit niedriger Aktivität mit dem Wildtyp verglichen werden (Abbildung S26a). R185‐Varianten außer R185K involvieren darüber hinaus eine extensive Fluktuation zwischen den ^4^ *C*_1_‐ und ^1^ *C*_4_‐Konformationen des Zuckerrings (Abbildung S26b–f), wodurch sich eine strukturelle Begründung für die Decarboxylierung als hauptsächlicher Reaktionsweg für die UDP‐GlcA‐Umsetzung durch diese Enzyme ergibt. Im Gegensatz dazu wird eine Konformationsänderung ^4^ *C*_1_ zu ^1^ *C*_4_ in der 4‐Ketohexuronsäure, und eine damit verbundene Reorientierung der Carboxylatgruppe von äquatorial zu axial, in MD‐Simulationen des **I^ROT^**‐Konformers des Wildtyps nicht beobachtet.

Zuletzt untersuchten wir den wichtigen Vorschlag aus der Evidenz über die R185‐Varianten, dass Decarboxylierung in dem Ausmaß vonstatten geht, in dem Bindetasche‐Interaktionen mit der Carboxylatgruppe im gegensätzlichen Rotationsisomer des verwendeten Substrats zerstört wurden. Wenn UDP‐GalA als Substrat angeboten wurde, zeigten Enzymvarianten, die das **I**‐Konformer beeinträchtigen (T126A, S127A), tatsächlich ein geringes Maß an Decarboxylierung (Tabelle 1; Abbildung S27), welches in ihren Reaktionen mit UDP‐GlcA eindeutig abwesend war. Im Gegensatz dazu ergab R185H Decarboxylierung in ≈2‐fach geringerer Menge und setzte relativ (≈2‐fach) mehr epimerisiertes Produkt in der Reaktion mit UDP‐GalA frei als in der Reaktion mit UDP‐GlcA (Tabelle 1; Abbildung S24). Die kompletten Zeitverläufe sind in den Hintergrundinformationen (Abbildungen S28–S30) gegeben.

## Zusammenfassung

**Figure 4:**
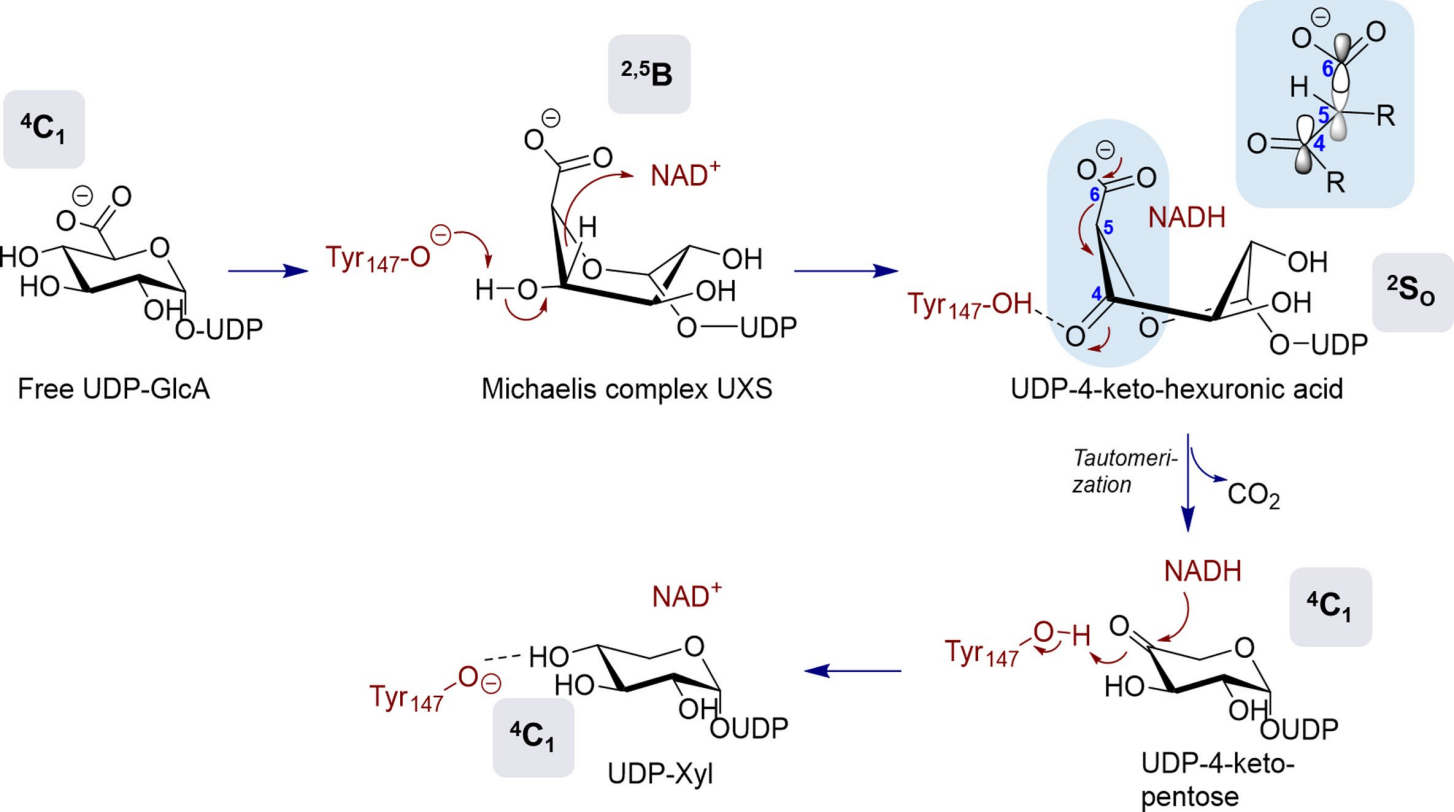
Vorgeschlagener Mechanismus von UXS in der Reaktion mit UDP‐GlcA zu UDP‐Xyl. Die Änderung der Ringkonformation von einem initialen ^4^ *C*_1_‐Sessel in UDP‐GlcA zu einem ^2^ *S*_O_‐Boot (skew‐boat) im UDP‐4‐Ketohexuronsäure‐Intermediat bringt die Carboxylatgruppe in eine axiale Orientierung und führt dadurch zu einer optimalen Orbitalausrichtung für eine rasche Decarboxylierung.[^6^, ^22^] Beachte Abbildung 1 für einen Vergleich mit UGAepi, welche stereoelektronische Kontrolle verwendet, um die Decarboxylierung zu verhindern.

Unsere Studie macht basierend auf Computerberechnungen und Experimenten jene koordinierten konformativen Änderungen (coupled motions) deutlich, die von BcUGAepi zur Repositionierung ihres UDP‐4‐Ketohexuronsäure‐Reaktionsintermediats verwendet werden, um eine selektive Epimerisierung zu erreichen. Wir decken dabei die Rolle von enzymunterstützten Distorsionen des Zuckerrings auf, welche steuernd für einen komplexen Rotationsweg wirken, der sich tiefgreifend von der einfachen Rotation eines Festkörpers unterscheidet. SDR‐Epimerasen, die Hexose/Pentose‐Nukleotid‐Substrate (z. B. UDP‐Galactose‐4‐Epimerase, UDP‐*N*‐Acetylglucosamin‐4‐Epimerase und andere) verwenden und daher 4‐Ketointermediate involvieren, die chemisch weniger vulnerabel sind als jenes der UGAepi, kommen nach geltenden Annahmen basierend auf der statischen Evidenz von Kristallstrukturen in ihrer Katalyse mit einer festkörperartigen Rotation (rigid‐body rotation) aus (Abbildung S31).[^3g^, ^3h^, ^17^, ^18^, ^19^, ^20^] Unsere Resultate legen UGAepi‐spezifische Strategien zur Vermeidung der Decarboxylierung der 4‐Ketohexuronsäure nahe. Erstens: Die Endpunkte der Rotation **I** und **I^ROT^** manifestieren die konformative Selektion des Enzyms für den Zweck der stereoelektronischen Kontrolle. Zweitens: Dynamische Reorganisation der Wasserstoffbrückenbindung mit der β‐Ketosäure‐Gruppe des Intermediats im Verlauf der koordinierten Bewegungen kann die Reaktivität des kurzlebigen Zuckerkonformers, das andernfalls leicht decarboxyliert werden würde, unterdrücken. Die Art der Handhabung der UDP‐4‐Ketohexuronsäure durch UGAepi zieht signifikantes, über die Epimerisierung hinausgehendes, mechanistisches Interesse auf sich, wenn man die Existenz einer eigenen SDR‐Familie an Decarboxylase‐Enzymen bedenkt.^[16]^ Die Reaktion dieser Decarboxylasen, am Beispiel der UDP‐Xylose‐Synthase (EC 4.1.1.35) verdeutlicht, involviert die genau gleiche aus Enzym‐NAD^+^ gebildete UDP‐4‐Ketohexuronsäure‐Spezies wie die UGAepi‐Reaktion, jedoch verläuft sie vom 4‐Ketointermediat ausschließlich über den Weg der Decarboxylierung (Abbildung 4).[^3e^, ^21^, ^22^]

Evidenz für eine Zuckerring‐Distorsion (^4^ *C*_1_ → ^2,5^ *B* und ^2^ *S*_O_) und damit einhergehende Positionsänderung des Carboxylats von äquatorial zu axial in der UXS‐gebundenen UDP‐GlcA sind konsistent mit stereoelektronischer Kontrolle durch das Enzym, nun jedoch aufgeboten, um die Decarboxylierung optimal voranzutreiben (Abbildung 4).^[22]^ Bemerkenswert ist daher, dass ortsgerichtete Substitutionen, welche mit der präzisen konformativen Auswahl in der BcUGAepi interferieren, die ursprüngliche Epimerase in einfache (“primitive”) UDP‐Xylose‐Synthasen umwandeln, die UDP‐GlcA in langsamen, jedoch völlig stereospezifischen Reaktionen decarboxylieren. Unsere Erkenntnisse stellen daher einen Zusammenhang zwischen konformativer Plastizität des Proteins und enzymatischer Reaktivität in der Konversion von UDP‐GlcA her. Unter Berücksichtigung der Diversität an Transformationen, die von SDR‐Enzymen an Zuckernukleotid‐Substraten katalysiert werden,[^10^, ^11^, ^12^, ^15^, ^16^, ^23^] erhält die Evidenz, dass Proteindynamik eine Rolle für den Erwerb einer spezifischen Enzymfunktion spielt, eine breite Relevanz im gesamten Kontext der Superfamilie. Ebenso kann sie praktische Relevanz in aktuell laufenden Anstrengungen für die Entdeckung und das Engineering von Enzymen in der angewandten Biokatalyse der Synthese von Zuckernukleotiden haben.[^14^, ^24^] Die Evidenz ist von mechanistischer Bedeutung, indem sie konformative Flexibilität mit der Selektion und der Kontrolle von Reaktionswegen verbindet. Sie stellt einen Beitrag zu einem wichtigen Feld der aktuellen Enzymforschung an der Wegkreuzung von Evolution, Engineering und Design dar.[^2f^, ^3a^, ^25^, ^26^]

## Experimentelles

Alle Details der verwendeten experimentellen und computergestützten Methoden sind in den Hintergrundinformationen gegeben.

## Interessenkonflikt

Die Autoren erklären, dass keine Interessenkonflikte vorliegen.

1

## Supporting information

As a service to our authors and readers, this journal provides supporting information supplied by the authors. Such materials are peer reviewed and may be re‐organized for online delivery, but are not copy‐edited or typeset. Technical support issues arising from supporting information (other than missing files) should be addressed to the authors.

Supporting Information

Supporting Information

## Data Availability

Die Daten, die die Ergebnisse dieser Studie unterstützen, sind auf begründete Anfrage beim Autor erhältlich.
