# Supplementary material for: Enzymatische C4‐Epimerisierung von UDP‐Glucuronsäure: präzise gesteuerte Rotation eines transienten 4‐Ketointermediats für eine invertierende Reaktion ohne Decarboxylierung
Source: Angew Chem Weinheim Bergstr Ger. 2022 Dec 15;135(4):e202211937. doi: 10.1002/ange.202211937 (PMC10952283; doi:10.1002/ange.202211937)
Supplement: Supplementary file 1 — Supporting Information [file ANGE-135-0-s001.pdf]

Angewandte Chemie
Eine Zeitschrift der Gesellschaft Deutscher Chemiker

Supporting Information

Enzymatische C4-Epimerisierung von UDP-Glucuronsäure: präzise gesteuerte Rotation eines transienten 4-Ketointermediats für eine invertierende Reaktion ohne Decarboxylierung

A. J. E. Borg, O. Esquivias, J. Coines, C. Rovira, B. Nidetzky**

Table of Contents

1. Experimental Procedures **3**

1.1. Computational Details 3

Modeling of the Enzyme Complex with NAD^+ and UDP-GlcA 3

Modeling of the Reaction of UDP-GlcA Oxidation to UDP-4-Keto-Hexuronic Acid 3

Modeling of the Rotation of UDP-4-Keto-Hexuronic Acid in the Enzyme Active Site 4

Modeling of Arg185 Variants in the Enzyme Complex with UDP-4-Keto-Hexuronic Acid 4

1.2. Experimental Details 5

Materials 5

Site-Directed Mutagenesis 5

Preparation of the BcUGAepi Variants 6

Activity Assays of BcUGAepi Variants 6

Product Burst Analysis in BcUGAepi Variants 6

R185H Reaction with Reduced Enzyme and Increased Substrate Concentration 6

Reduction of UDP-4-Keto-Pentose to UDP-Xylose by BcUGAepi Wild Type 7

Reaction with Addition of UDP-Xylose 7

HPLC 7

Product Identification by ^{1}H-NMR 7

2. Supporting Figures **8**

3. Supporting Tables **39**

4. Supporting Movie Caption **40**

5. References **40**

6. Author Contributions **41**

1. Experimental Procedures

1.1. Computational Details

Modeling of the Enzyme Complex with NAD^+ and UDP-GlcA
The initial structure for the Michaelis complex (MC) was taken from the X-ray crystal structure of BcUGAepi in complex with UDP-GlcA and NAD^+ (PDB entry 6ZLD, at 1.80 Å resolution).[1] Both subunits of the homodimer were considered. Protonation states of titratable residues were assigned using Propka3,[2] as well as analysis of the environment of each residue, considering a pH value of 7.6. The carboxylate group of GlcA and the hydroxyl group of Y149 were considered as deprotonated according to the measured pK_a values of the catalytic residues in SDR enzymes,[3] as well as previous work on related SDRs.[4,5] The enzyme complex was solvated in a cubic cell (96.129 × 113.404 × 115.233 Å^3) with 32658 water molecules and 8 Na^+ ions to compensate the charge of the system, using the Leap module of Amber18.[6]

The following force field parameter sets were used for the classical molecular dynamics (MD) simulations: FF14SB,[7] GLYCAM06,[8] GAFF2[6] and TIP3P[9] for the protein, the monosaccharide, UDP, and the water molecules respectively. NAD^+ parameters were taken from Bryce group database.[10] Atomic partial charges (RESP) of Tyr149 and UDP were calculated at the HF/6-31G* level of theory with Gaussian09.[11] MD simulations were performed using Amber18.[6] The following protocol was used. First, the energy was minimized by fixing the enzyme, the substrate and the cofactor, followed by energy minimization of the whole system. Afterwards, the system was heated up to 300 K in the NVT ensemble. Weak spatial constraints were applied to the protein, the substrate and the cofactor during the first 200 ps of the simulation, while the system was heated to 100 K. Two more ramps of 100 K and 100 ps were applied. Once the target temperature of 300 K was reached, the simulation was extended in the NVT ensemble for additional 100 ps, followed by 1 ns in the NPT ensemble to equilibrate the density, using a timestep of 2 fs along with the SHAKE algorithm.[12] Weak restraints at the reactive distances were used to maintain the substrate in a reactive position during the heating and density equilibration phases. Restraints were gradually released during the first 10 ns of the production phase, consisting in three replicas of 200 ns (NPT ensemble). Figure S1b shows the evolution of the RMSD backbone during the simulation. All classical MD simulations reported in this work followed the same protocol.
The occurrence of reactive configurations was measured by analysing the distances among the atoms involved in covalent bond formation or cleavage (O_{Tyr}-H4 and C4'-H). We observed that two different states (MC and MC') were sampled in classical MD, as depicted in Figure S1a. One MD snap-shot corresponding to the most reactive configuration (MC) was taken to initiate the QM/MM simulations.

Modeling of the Reaction of UDP-GlcA Oxidation to UDP-4-Keto-Hexuronic Acid
The simulations were performed using the method developed by Laio *et al.*,[13] which combines Car–Parrinello MD,[14] based on Density Functional Theory (DFT), with force-field MD, as implemented in the CPMD code.[15] In this approach, the system is partitioned into quantum mechanics (QM) and molecular mechanics (MM) fragments. The dynamics of the atoms on the QM fragment depend on the electronic density, ρ(r), computed with DFT, whereas the dynamics of the atoms on the MM fragment are ruled by an empirical force field. The QM/MM interface is modeled by the use of link-atom that saturates the QM region. The electrostatic interactions between the QM and MM regions were handled via a fully Hamiltonian coupling scheme,[16] where the short-range electrostatic interactions between the QM and the MM regions are explicitly taken into account for all atoms. An appropriately modified Coulomb potential is used to ensure that no unphysical escape of the electronic density from the QM to the MM region occurs. The electrostatic interactions with the more distant MM atoms are treated via a multipole expansion. Bonded and van der Waals interactions between the QM and the MM regions were treated with the standard AMBER force-field. Long-range electrostatic interactions between MM atoms were described with the P3M implementation,[14] using a 64 x 64 x 64 mesh. The QM region was selected as to include the sidechain of Y149, T126, the GlcA sugar and the phosphate groups of UDP, and the nicotinamide moiety and the phosphate groups of NAD^+. Four hydrogen link atoms were employed to saturate the QM atoms that have dangling bonds due to the separation between the QM and MM regions. The QM region (101 atoms) was enclosed in a box of dimensions 21.969 × 21.392 × 18.669 Å^3. The radii of the layers of the electrostatic coupling scheme were set at 15 a.u. (NN), 21 a.u. (MIX) and 29 a.u. (ESP).

The electronic subsystem was described with DFT using the Perdew–Burke-Ernzerhof (PBE) functional,[13] which has proven to give good results for carbohydrate conformations.[17] A plane-wave basis with a kinetic energy cutoff of 70Ry was used for the expansion of Kohn-Sham orbitals. This setup has been demonstrated to be suitable for reaction mechanisms in carbohydrate-active enzymes and carbohydrate conformations.[15–17] Norm-conserving Troullier–Martins *ab initio* pseudopotentials[18] were used for all elements. A fictitious electron mass of 600 a.u. was used for the Car-Parrinello Lagrangian and the simulations used an integration time step of 5 a.u. (≈ 0.12 fs). The temperature and the electron kinetic energy was controlled with two Nosé-Hoover thermostats[18] at 300 K and 0.014 H., respectively. The structure was optimized by annealing of the atomic velocities and a QM/MM MD simulation for 4 ps was subsequently performed. A similar setup was previously used in the modeling chemical reactions in carbohydrate-active enzymes.[19]

The free energy landscape (FEL) of the oxidation reaction was explored by QM/MM metadynamics with two collective variables (CVs), using the metadynamics driver provided by the Plumed2 plugin (version 2.3.3).[20] Each CV was taken as a difference between two distances that need to be broken/formed during the reaction. The first CV measures the transfer of the proton between Y149 and the sugar 4-OH [CV_1 = (O_{Y149} ···H4) – (O4-H4)]. The second CV measures the interaction between the sugar C4-H hydrogen atom and the nicotinamide moiety of NAD^+ [CV_2 = (C4'···H) – (C4-H)]. Thus, the second CV accounts for the transfer of the H atom between the sugar and the nicotinamide. The width of the biasing Gaussian functions and the deposition time were selected according to the oscillations of each CV in an unbiased QM/MM MD simulation (0.2 Å for the Gaussian width, for both CVs, and deposition time of 300 MD steps). The hills height was set to 1.2 $kcalmol^{-1}$ during the whole simulation, but decreased to 0.6 $kcalmol^{-1}$ when the system was about to cross over the TS. The simulation was stopped after one re-crossing over the TS, as recommended for chemical reactions.[21] A total number of 1362 Gaussians were deposited (in terms of simulation time, this corresponds to 50 ps). The evolution of the CVs along the metadynamics simulation is shown in Figure S33. Trajectories were analyzed with cpptraj (from Amber18),[6] Plumed2,[20] VMD,[16] MEPSAND[22] and in house python3 scripts.

Modeling of the Rotation of UDP-4-Keto-Hexuronic Acid in the Enzyme Active Site

The dynamics of the 4-keto-hexuronic acid in the active site of BcUGAepi was modeled by classical metadynamics, starting from the structure of the system after the oxidation step (state **I**). The force field parameters for the 4-keto-hexuronic acid moiety were taken as follows. Bond and angle parameters were taken from GLYCAM06[8] and GAFF2[6]. Parameters for dihedral angles, which are not specifically defined in GLYCAM06, were obtained by performing relaxed scan calculations at the MP2/6-31* level of theory. The results of the scan for each dihedral angle were fitted to an MM energy profile using the force field toolkit VMD plugin[23] (dihedral angle parameters are given in Table S2). The remaining force-field parameters (protein residues, water, NADH and UDP) were the same as in the simulations of the MC complex

An MD simulation for 200 ns of state **I** was performed to accommodate the enzyme to the UDP-4-keto-hexuronic acid substrate. Afterwards, rotation of 4-keto hexuronic acid was activated by metadynamics, using two collective variables (Figure S3). The first one was taken as a dihedral angle that accounts for the rotation of the ring plane around a parallel axis to the ring plane. The second CV was taken as the difference between two distances: the distance from R185 to sugar carboxylate (CZ_{R185} – $C6_{sugar}$) and the distance from R185 to the geometrical center of the sugar ring (CZ_{R185} – sugar ring center). The height and widths of the Gaussian functions were taken as 0.1 kcal/mol and 0.1 Å for both CVs, respectively, and Gaussian functions were deposited every 500 MD steps. A total number of 8000 Gaussians were deposited (8 ns in terms of simulation time). The simulation drove the 4-keto-hexuronic acid intermediate (**I**) to a conformation in which the GlcA ring is fully rotated around the longitudinal axis and the carboxylate group interacts with R185 (state $\mathbf{I^{ROT}}$). The metadynamics simulation was stopped after filling the products well, since re-crossing over the TS did not occur in the timescale of the simulation. However, an additional metadynamics simulation starting from state $\mathbf{I^{ROT}}$, using the same CVs, successfully brought the system backwards towards **I**. This experiment was repeated six times for each state (**I** and $\mathbf{I^{ROT}}$) (Table S3) to have a better estimate of the rotation energy barrier and the energy difference between **I** and $\mathbf{I^{ROT}}$. Additional MD simulations starting from the products complex (enzyme complex with NAD^+ and UDP-GalA, PDB entry 6ZLL) after converting the GalA sugar into 4-keto-hexuronic acid led to a very similar structure as $\mathbf{I^{ROT}}$ (Figure S34), which validates the structure obtained from the metadynamics simulations.

Modeling of Arg185 Variants in the Enzyme Complex with UDP-4-Keto-Hexuronic Acid

Models for R185 variants were built from the crystal structure of the enzyme complex with UDP-GalA and NAD^+ (PDB entry 6ZLL)[1] after manual change of the GalA moiety to 4-keto-hexuronic acid and assigning the suitable protonation state to the cofactor and all protein residues. Replacement of R185 by histidine, lysine, aspartic acid or alanine was done using the Leap module of Amber18.[6] Arg, His or Asp in position 185 were considered as protonated, since there are two phosphate groups with negative charges close to it. Additional water molecules were added to fill the space left by the smaller residues and sodium ions were added to achieve neutrality of the simulated system. Classical MD simulations (20 ns equilibration and 200 ns production) were performed for each complex, following the same procedure as in the simulations of the MC complex.

1.2. Experimental Details

Materials

The synthetic gene of BcUGAepi was ordered in pET17b-expression vector (pET17b_BcUGAepi) from GenScript (USA). UDP-D-glucuronic acid (UDP-GlcA; >98% purity) and sodium pyruvate were from Sigma-Aldrich (Vienna, Austria). NAD^+ (>98% purity) was from Roth (Karlsruhe, Germany). Deuterium oxide (99.96% ^2H) was from Euriso-Top (Saint-Aubin Cedex, France). UDP-xylose and UDP-4-keto-pentose were synthesized according to protocols from literature.[24,25] All other reagents and chemicals were of highest available purity. For plasmid DNA isolation, the GeneJET Plasmid Miniprep Kit (Thermo Scientific; Waltham, MA, USA) was used. DpnI and Q5® High-Fidelity DNA polymerase were from New England Biolabs (Frankfurt am Main, Germany) and D-lactate dehydrogenase from Megazyme (Vienna, Austria). All other enzymes were prepared in-house. Oligonucleotide primers were from Sigma-Aldrich (Vienna, Austria). *E. coli* NEB5α competent cells were from New England Biolabs (Frankfurt, Germany). *E. coli* Lemo21(DE3) cells were prepared in-house.

Site-Directed Mutagenesis

BcUGAepi variants were prepared using a modified QuikChange protocol. PCRs were carried out in the reaction volume of 50 µL using 20 ng of plasmid DNA as template and 0.2 µM of forward or reverse primer. Q5 DNA polymerase was used for DNA amplification. The sequences of DNA oligonucleotide primers used for the mutagenesis in BcUGAepi are shown below. The underlined nucleotides highlight the mutations introduced by PCR.

Name	Mutation	DNA primer sequence (5'-3')
T126A_fw<br>T126A_rv	T126A	CAAGTTTATCCACATTAGCGCAAGCAGCGTGTACGGCGAG<br>CTCGCCGTACACGCTGCTTGCGCTAATGTGGATAAACTTG
T178A_fw<br>T178A_rv	T178A	GATCCTGCGTTACTTTGCCGTTTATGGTCCG<br>CGGACCATAAACGGCAAAGTAACGCAGGATC
S127A_fw<br>S127A_rv	S127A	GTTTATCCACATTAGCACCAGCAGCGTGTACGGCGAGAAG<br>CTTCTCGCCGTACACGCTGCTGGTGCTAATGTGGATAAAC
S128A_fw<br>S128A_rv	S128A	CACATTAGCACCAGCGCAGTGTACGGCGAGAAG<br>CTTCTCGCCGTACACTGCGCTGGTGCTAATGT
S128E_fw<br>S128E_rv	S128E	CACATTAGCACCAGCGAAGTGTACGGCGAGAAG<br>CTTCTCGCCGTACACTTCGCTGGTGCTAATGT
R185A_fw<br>R185A_rv	R185A	TATGGTCCACGTCAGGCACCGGACATGGCGTTC<br>GAACGCCATGTCCGGTGCCTGACGCGGACCATA
R185D_fw<br>R185D_rv	R185D	TATGGTCCGCGTCAGGATCCAGACATGGCGTTC<br>GAACGCCATGTCTGGATCCTGACGCGGACCATA
R185H_fw<br>R185H_rv	R185H	TATGGTCCACGTCAGCACCCGGACATGGCGTTC<br>GAACGCCATGTCCGGGTGCTGACGTGGACCATA
R185K_fw<br>R185K_rv	R185K	TATGGTCCGCGTCAGAAACCGGACATGGCGTTC<br>GAACGCCATGTCCGGTTTCTGACGCGGACCATA

First, three cycles of linear PCR amplification were performed with separate forward and reverse primers (initial denaturation: 30 sec/98 °C; 3 cycles amplification: 10 sec/98 °C for denaturation, 15 sec/55 °C for primer annealing and 6 min/72 °C extension; final extension: 5 min/72 °C). The reaction mixtures were combined to mix the forward and reverse primer solutions, divided again into two mixtures of 50 µL (for better heat transfer) and the PCR program was restarted for 15 cycles of exponential DNA amplification (initial denaturation: 30 sec/98 °C; 3 cycles amplification: 10 sec/98 °C for denaturation, 15 sec/55 °C for primer annealing and 6 min/72 °C extension; final extension: 5 min/72 °C). Residual template DNA was removed by addition of 10 U DpnI and incubation at 37 °C for 16 h. DpnI was inactivated by incubating at 80 °C for 20 min, the mixtures were centrifuged and the PCR products analyzed by agarose gel electrophoresis and visualized by DNA staining. The PCR products were directly transformed into chemically competent *E. coli* NEB5α cells (New England Biolabs). Plasmid DNA was extracted and sequenced with T7prom/T7term primers provided by LGC Genomics (Berlin, Germany) to confirm the mutations. The correct construct was transformed into *E. coli* Lemo21(DE3) cells followed by expression of BcUGAepi.

Preparation of the BcUGAepi Variants

The site-directed amino acid substitutions were introduced by a modified QuikChange protocol and the successful mutagenesis confirmed by DNA sequencing (LGC Genomics, Berlin, Germany). All BcUGAepi variants were produced by expression in *E. coli* Lemo21(DE3) cells that harbored the pET17b-expression vector containing the gene of interest. The proteins were purified utilizing the C-terminal Strep-tag, and the size and purity of the proteins were confirmed by SDS-PAGE. The Strep-tag columns were washed thoroughly with extreme caution after each purification, to eliminate any chance of cross-contamination in between the mutants. Full details of the expression and purification conditions are reported elsewhere.[26]

Activity Assays of BcUGAepi Variants

The reaction mixtures (250 µL final volume) of the BcUGAepi variants contained 1 mM UDP-GlcA (or UDP-GalA) and 100 µM NAD^+ in sodium phosphate buffer (50 mM Na_2HPO_4, 100 mM NaCl, pH 7.6). The enzyme concentrations were variant-dependent: 0.07 $mgmL^{-1}$ (1.9 µM, wild type), 5 $mgmL^{-1}$ (135 µM, S128E), 10 $mgmL^{-1}$ (270 µM, T126A; R185A), 1.6 $mgmL^{-1}$ (43 µM, T178A), 0.2 $mgmL^{-1}$ (5.4 µM, S127A; S128A), 4.7 $mgmL^{-1}$ (127 µM, R185K), 15 $mgmL^{-1}$ (405 µM, R185D; R185H). The reactions were incubated at 23 °C, quenched with methanol (50% (v/v) final concentration) at desired time points and the precipitated enzyme removed by centrifugation (16100 g, 4 °C, 30 min) prior to HPLC analysis. The initial rates were determined from the linear part of the time-course by dividing the slope of the linear regression ($mMmin^{-1}$) by the enzyme concentration ($mgmL^{-1}$) giving the initial rate in µmol(min mg protein)$^{-1}$. The product burst activity of R185H, R185D and R185K variants was calculated from the time point of 1 min (Figures S20, S21, S22) by dividing the total amount of products (in mM) by the enzyme concentration used (in $mgmL^{-1}$).

Product Burst Analysis in BcUGAepi Variants

General Analysis

The burst phase generally refers to the fast initial product release in the overall reaction time course recorded by HPLC. The reaction conditions are those shown above under Activity Assays of BcUGAepi variants. Note: the sampling (1.0 min of incubation) may not have been suitable in all cases to obtain the product release within the first turnover of the enzyme.

R185H Reaction with Reduced Enzyme and Increased Substrate Concentration

R185H variant reaction was performed with 100-fold decreased enzyme concentration and 10-fold increased UDP-GlcA concentration. The reaction mixture (100 µL final volume) contained 10 mM UDP-GlcA, 1 mM NAD^+ and 0.15 $mgmL^{-1}$ (4.05 µM) R185H enzyme in sodium phosphate buffer (50 mM Na_2HPO_4, 100 mM NaCl, pH 7.6). The reaction was incubated at 23 °C and sampled over 5 days (sampling as described under “Activity Assays of BcUGAepi Variants”). The samples 0-15 min were used for the calculation of the burst activity and the activity after the burst was calculated from the time points 40-7200 min (Figure S24). The activities were determined by dividing the slope of the linear regression ($mMmin^{-1}$) by the enzyme concentration ($mgmL^{-1}$) giving the rate in µmol(min mg protein)$^{-1}$.

Reduction of UDP-4-Keto-Pentose to UDP-Xylose by BcUGAepi Wild Type

To investigate the role of UDP-xylose in the product burst, the reduction of UDP-4-keto-pentose to UDP-xylose was examined by wild type BcUGAepi. The reaction mixture (250 µL final volume) contained 1 mM UDP-4-keto-pentose, 10 mM NADH and 8 $mgmL^{-1}$ (216 µM) wild type enzyme in sodium phosphate buffer (50 mM Na_2HPO_4, 100 mM NaCl, pH 7.6). The reaction was incubated at 23 °C and sampled over 12 h (sampling as described under “Activity Assays of BcUGAepi Variants”). The product burst activity at 1 min (Figure S25a) was calculated by dividing the total amount of products (in mM) by the enzyme concentration used (in $mgmL^{-1}$). The activity after the burst was determined by dividing the slope of the linear regression ($mMmin^{-1}$) by the enzyme concentration ($mgmL^{-1}$) giving the rate in µmol(min mg protein)$^{-1}$.

Reaction with Addition of UDP-Xylose

The reaction mixture (250 µL final volume) contained 10 mM UDP-4-keto-pentose, 100 µM UDP-xylose, 10 mM NADH and 1 $mgmL^{-1}$ (27 µM) wild type BcUGAepi in sodium phosphate buffer (50 mM Na_2HPO_4, 100 mM NaCl, pH 7.6). UDP-xylose was mixed with the reaction buffer, UDP-4-keto-pentose and NADH prior to enzyme addition. The reaction was incubated at 23 °C and sampled over 19 h (sampling as described under “Activity Assays of BcUGAepi Variants”). The initial rate was calculated from the linear part of the time-course (0-15 min, Figure S25b) by dividing the slope of the linear regression ($mMmin^{-1}$) by the enzyme concentration ($mgmL^{-1}$) giving the rate in µmol(min mg protein)$^{-1}$.

HPLC

The sugar nucleotides and NAD^+/NADH were separated with Shimadzu Prominence HPLC-UV system (Shimadzu, Korneuburg, Austria) on a Kinetex C18 column (5 µm, 100 Å, 50 x 4.6 mm) using an isocratic method with 5% acetonitrile and 95% tetrabutylammonium bromide buffer (40 mM TBAB, 20 mM K_2HPO_4/KH_2PO_4, pH 5.9) as mobile phase. UDP-sugars, NAD^+ and NADH were detected by UV at 262 nm wavelength. The amount of UDP-GlcA/UDP-GalA/UDP-Xyl/UDP-4-keto-pentose formed was determined based on the relative integrated peak areas. Due to the close proximity of the peaks of UDP-Xyl and UDP-4-keto-pentose, standards of isolated compounds were used as reference for the retention times.[24]

Product Identification by ^{1}H-NMR

The absence of UDP-arabinose in R185 variants’ reactions was confirmed by NMR. Due to the poor activity of these variants, standard sample preparation for the measurements was not possible (except for the R185A variant). The enzymatic activity in D_2O was too low to detect any product formation on NMR. Performing the reactions in H_2O, freeze-drying the samples and re-dissolving in D_2O prior to the NMR measurements was also tried, but the signals from the excess of the remaining substrate (UDP-GlcA) were overlapping with the signals of the products. To improve the resolution of the NMR spectra and to make the product signals detectable in R185H, R185K and R185D reactions, half of the remaining substrate was enzymatically converted to UDP-4-keto-pentose by ArnA (expression and purification of ArnA are reported elsewhere[26]) prior to the measurements. The reaction mixtures contained 2 mM UDP-GlcA and 100 µM NAD^+ in sodium phosphate buffer (50 mM Na_2HPO_4, 100 mM NaCl, pH 7.6). The enzyme concentrations were 6 $mgmL^{-1}$ (162 µM, R185A), 0.9 $mgmL^{-1}$ (24.3 µM, R185D), 14.7 $mgmL^{-1}$ (397 µM, R185H) and 7.7 $mgmL^{-1}$ (208 µM, R185K). After 24 h at 23 °C, the progress of the reaction was confirmed on HPLC and the following components were added: 1 $mgmL^{-1}$ ArnA, 10 mM sodium pyruvate, 20 UmL^{-1} D-lactate dehydrogenase. The reaction was run for 30 min and followed on HPLC, and the enzymes removed with Vivaspin Turbo centrifugal filter tubes (10 kDa cut-off, Sartorius) when ~50% of UDP-GlcA was converted to UDP-4-keto-pentose. The addition of ArnA into the R185A reaction was not necessary, due to the better conversion of UDP-GlcA in this reaction (higher stability of R185A variant allowed to use 30 °C instead of 23 °C reaction temperature, resulting in more substantial substrate depletion). The reaction mixtures were lyophilized (Christ Alpha 1-4 lyophilizer, B. Braun Biotech International, Melsungen, Germany) and the obtained solid material re-dissolved in 600 µL of D_2O for the NMR measurements. The acquisition was carried out on a Varian INOVA 500-MHz NMR spectrometer (Agilent Technologies, Santa Clara, California, USA). The VNMRJ 2.2D software was used for the measurements. ^{1}H-NMR spectra (499.98 MHz) were recorded on a 5 mm indirect detection PFG-probe with pre-saturation of the water signal by a shaped pulse. The spectra were analyzed using MestReNova 16.0 (Mestrelab Research, S.L.). The products were identified based on their chemical shifts, by using reference values from literature.[24]

2. Supporting Figures

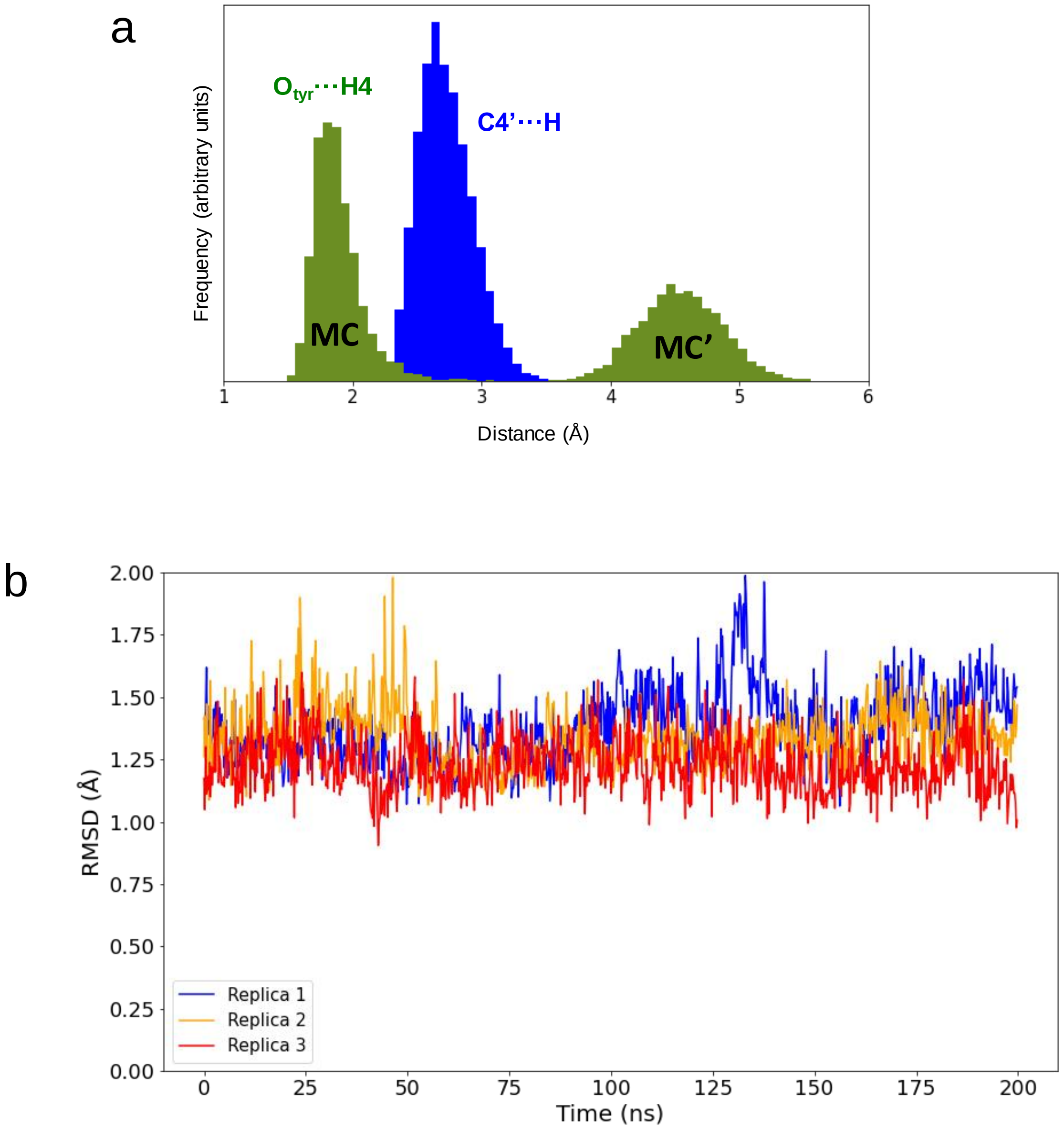

Figure S1. Classical MD simulations of BcUGAepi in complex with NAD^+ and UDP-GlcA. **a**. Histogram of the values of the main catalytic distances for proton transfer (O_{Tyr149}···H4) and hydride transfer (C4-H_{NAD}). According to the distance between O_{Tyr149} and H4, two states are explored. These states correspond to the MC and MC' states that were subsequently explored in QM/MM simulations as well. **b**. RMSD of the protein backbone along the production simulation.

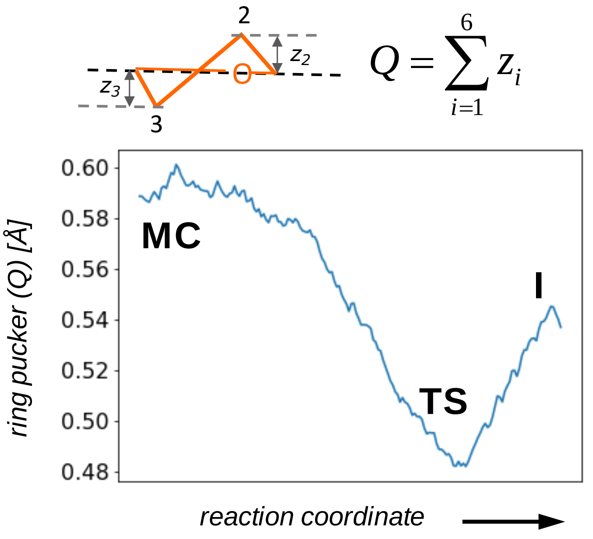

Figure S2. Evolution of the Q ring puckering coordinate (the amplitude of the Cremer-Pople sphere)[27] along the reaction coordinate of the oxidation reaction.

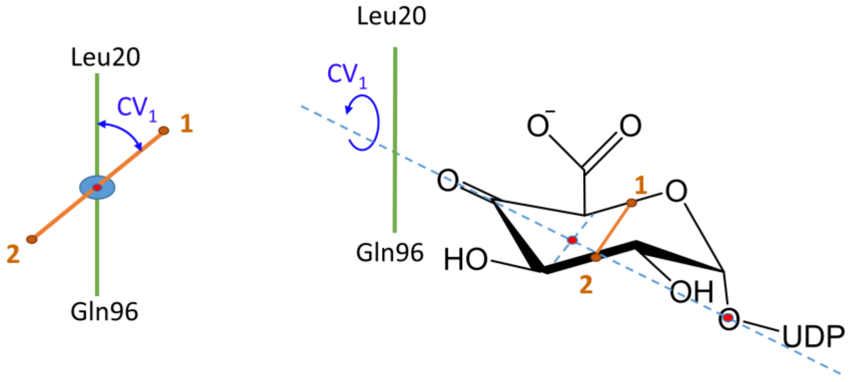

Figure S3. Collective variables used in the metadynamics simulations of rotation of the 4-keto-hexuronic acid moiety in the active site of BcUGAepi. CV_1 is a dihedral angle that measures the rotation of the 4-keto-hexuronic acid with respect to the protein. The rotation axis connects the glycosidic oxygen and the geometrical center between C4 and C5. The dihedral angle is defined by the rotation axis and the following two vectors. The first vector (orange line) connects the center of the C2-C3 bond with the center of the C5-O5 bond. The second vector (green line) connects the two residues located above and below the sugar (Leu20 and Gln96, respectively) that move little during the rotation process. The center of mass of the backbone atoms of each residue is considered. CV_2 (not shown in the picture) measures the proximity of the carboxylate group to Arg185. It was defined as the difference of two distances: the distance between carboxylate C atom (C6) and the center of the guanidinium group of Arg185 and the distance between the geometrical center of the sugar ring (X) and the guanidinium group CV_2 = ($C6\cdots C_{Arg185}$) – ($X\cdots C_{Arg185}$).

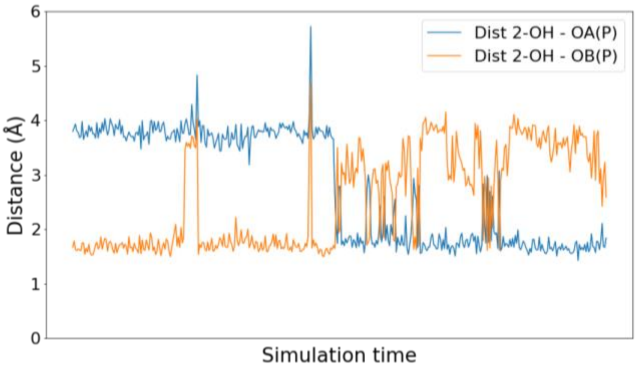

Figure S4. Distance between the 2-OH from the intermediate to the oxygens from β-phosphate of the UDP during metadynamics simulation of rotation of the 4-keto-hexuronic acid moiety.

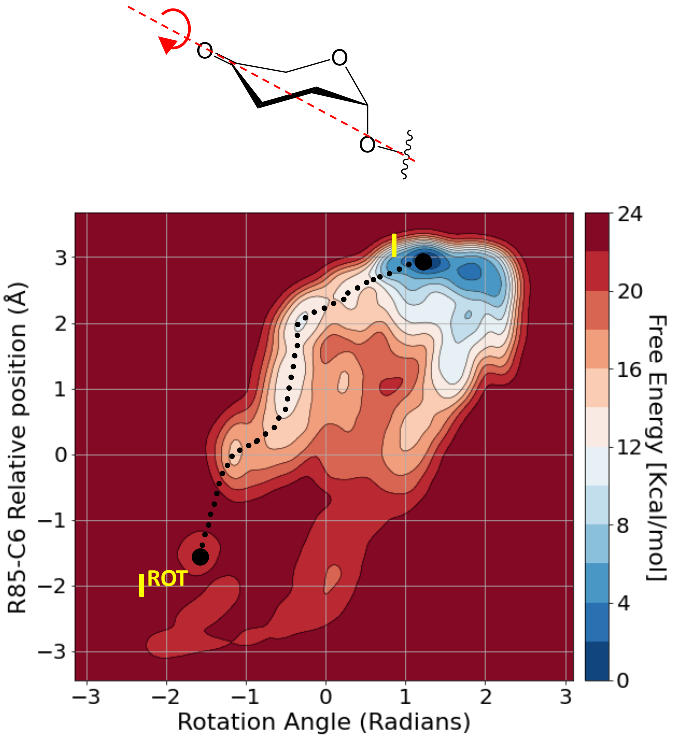

Figure S5. Rotational coordinate of the 4-keto-hexuronic acid in the active site of BcUGAepi obtained from metadynamics simulations from **I** to $\mathbf{I}^{\mathbf{ROT}}$, with the sugar restrained in the $^{4}C_{1}$ conformation. The simulation was stopped once the system reached the $\mathbf{I}^{\mathbf{ROT}}$ state. Therefore, only the free energy barrier from **I** to $\mathbf{I}^{\mathbf{ROT}}$ (> 20 kcal/mol) is meaningful.

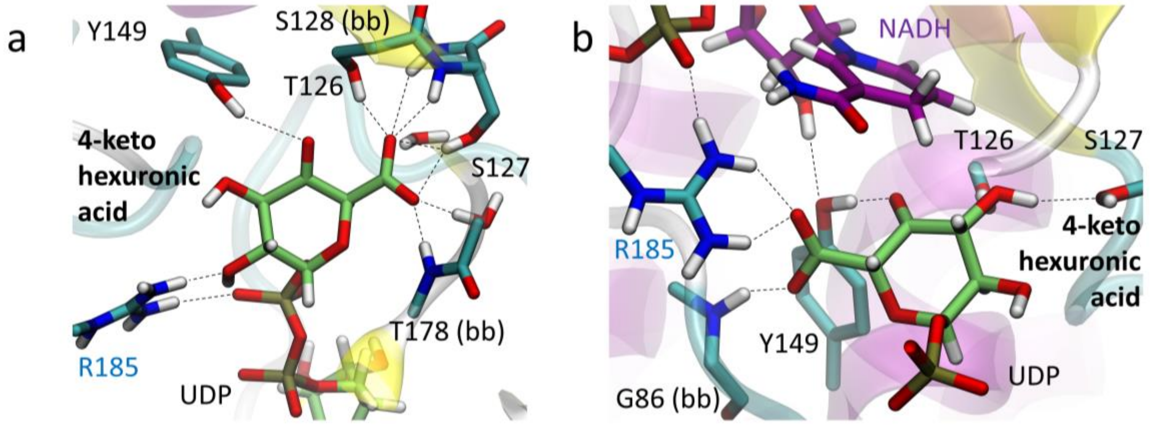

Figure S6. Interactions of sugar carboxylate in I and I^{ROT}. **a**. Representative snapshot of I. **b**. Representative snapshot of I^{ROT}, the perspective is rotated 90º in reference to **a** for better visualization of the interactions. bb = backbone, it is specified when the carboxylate interacts with the backbone of that residue.

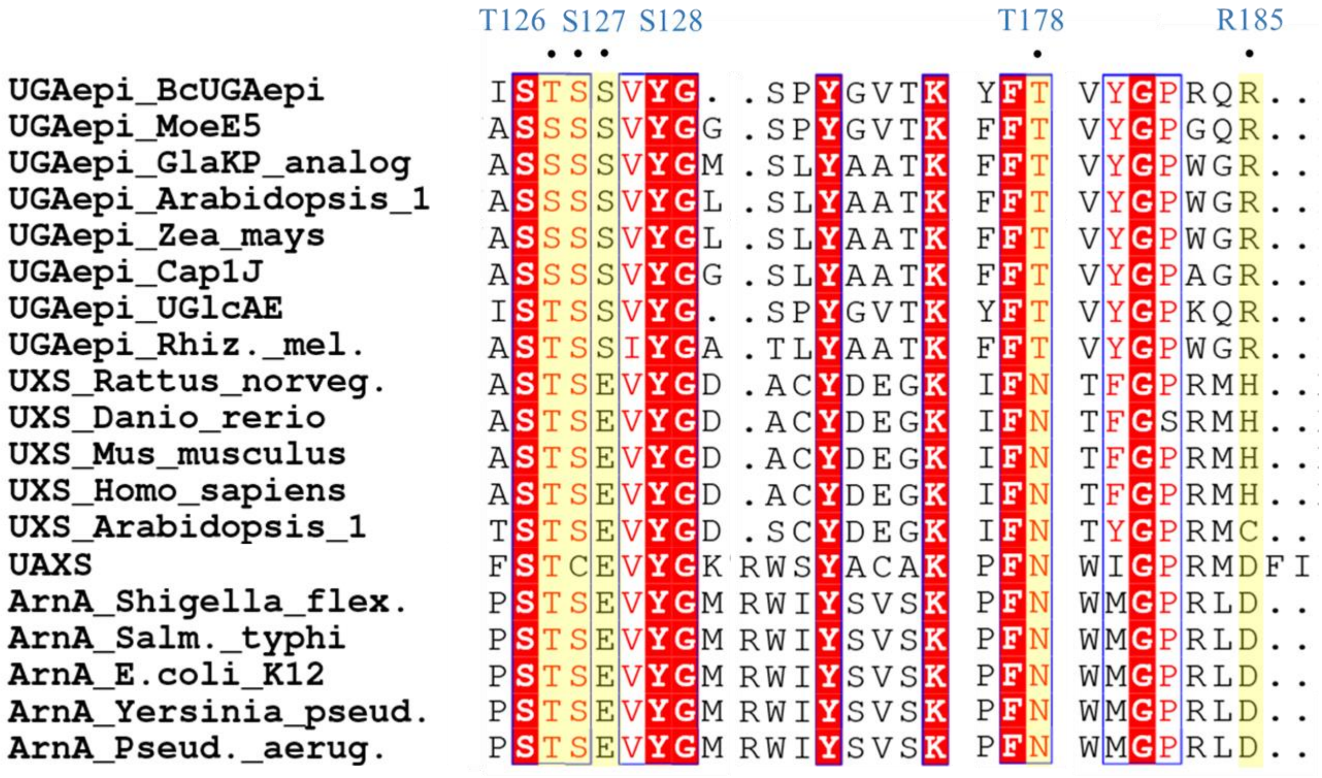

Figure S7. A partial multiple sequence alignment of UDP-GlcA decarboxylases (UAXS, UXS, ArnA) and UGAepis. The residues (Thr126, Ser127, Ser128, Thr178, Arg185) targeted by mutagenesis are highlighted in yellow.

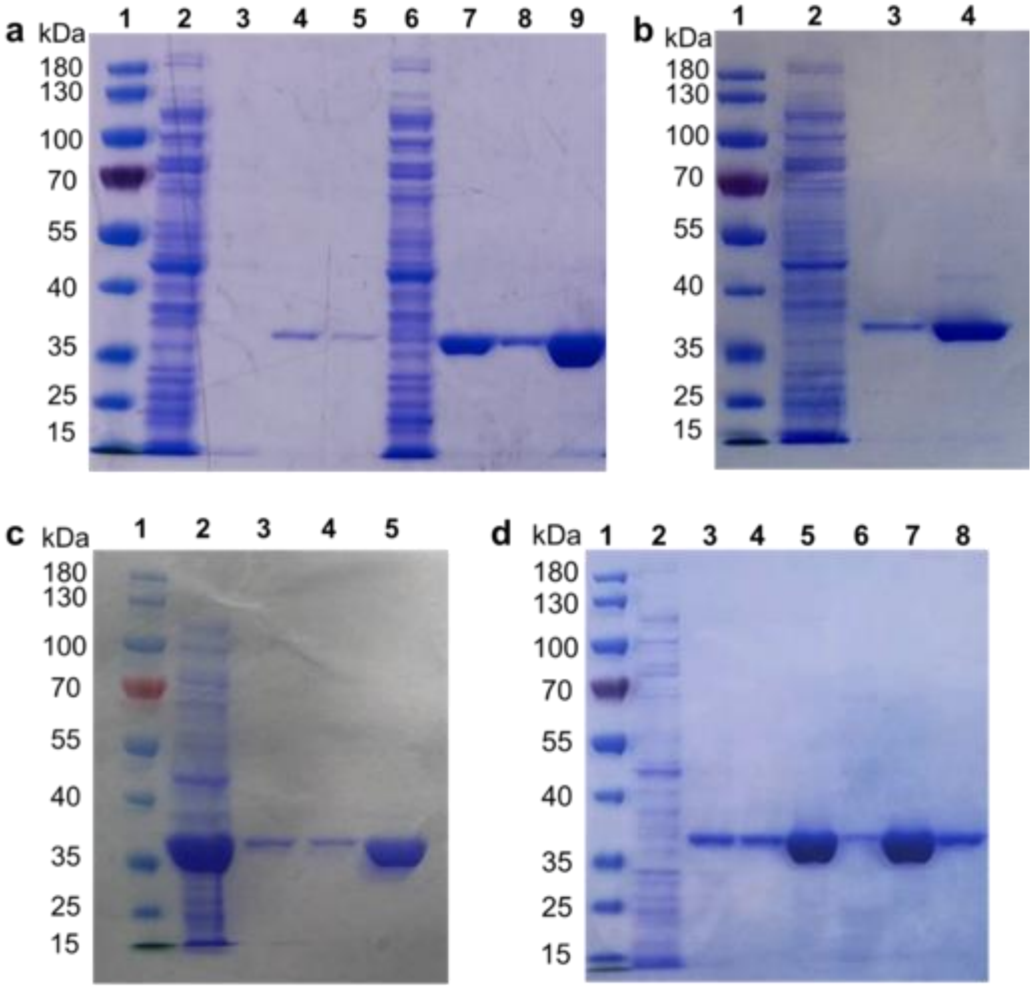

Figure S8. SDS-polyacrylamide gels from Strep-tag purifications of BcUGAepi variants (~37 kDa). **a**. The gel from the purification of the T178A and T126A variants. Lane 1: molecular mass ladder, lane 2: flow through fraction from purification of T178A, lane 3: washing fraction from purification of T178A, lane 4 and 5: elution fractions from purification of T178A, lane 6: flow through fraction from purification of T126A, lane 7: washing fraction from purification of T126A, lane 8 and 9: elution fractions from purification of T126A. **b**. The gel from the purification of the R185A variant. Lane 1: molecular mass ladder, lane 2: flow through fraction, lane 3: washing fraction, lane 4: elution fraction. **c**. The gel from the purification of the S128E variant. Lane 1: molecular mass ladder, lane 2: flow through fraction, lane 3: washing fraction, lane 4 and 5: elution fractions. **d**. The gel from the purification of the S127A and S128A variants. Lane 1: molecular mass ladder, lane 2: flow through fraction from purification of S127A, lane 3: washing fraction from purification of S127A, lane 4 and 5: elution fractions from purification of S127A, lane 6: washing fraction from purification of S128A, lane 7 and 8: elution fractions from purification of S128A.

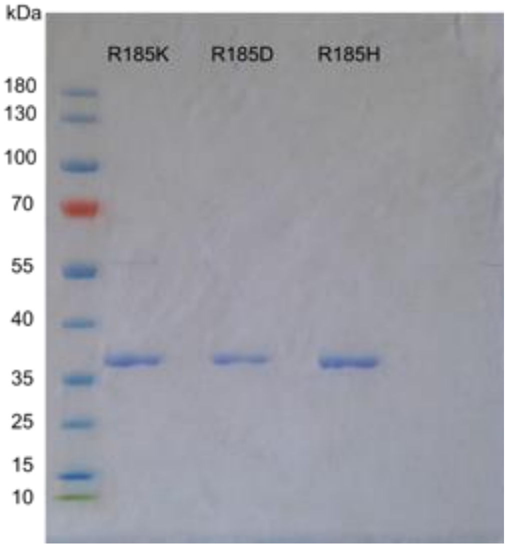

Figure S9. SDS-polyacrylamide gel of the elution fractions from the Strep-tag purifications of BcUGAepi variants R185K, R185D and R185H (~37 kDa).

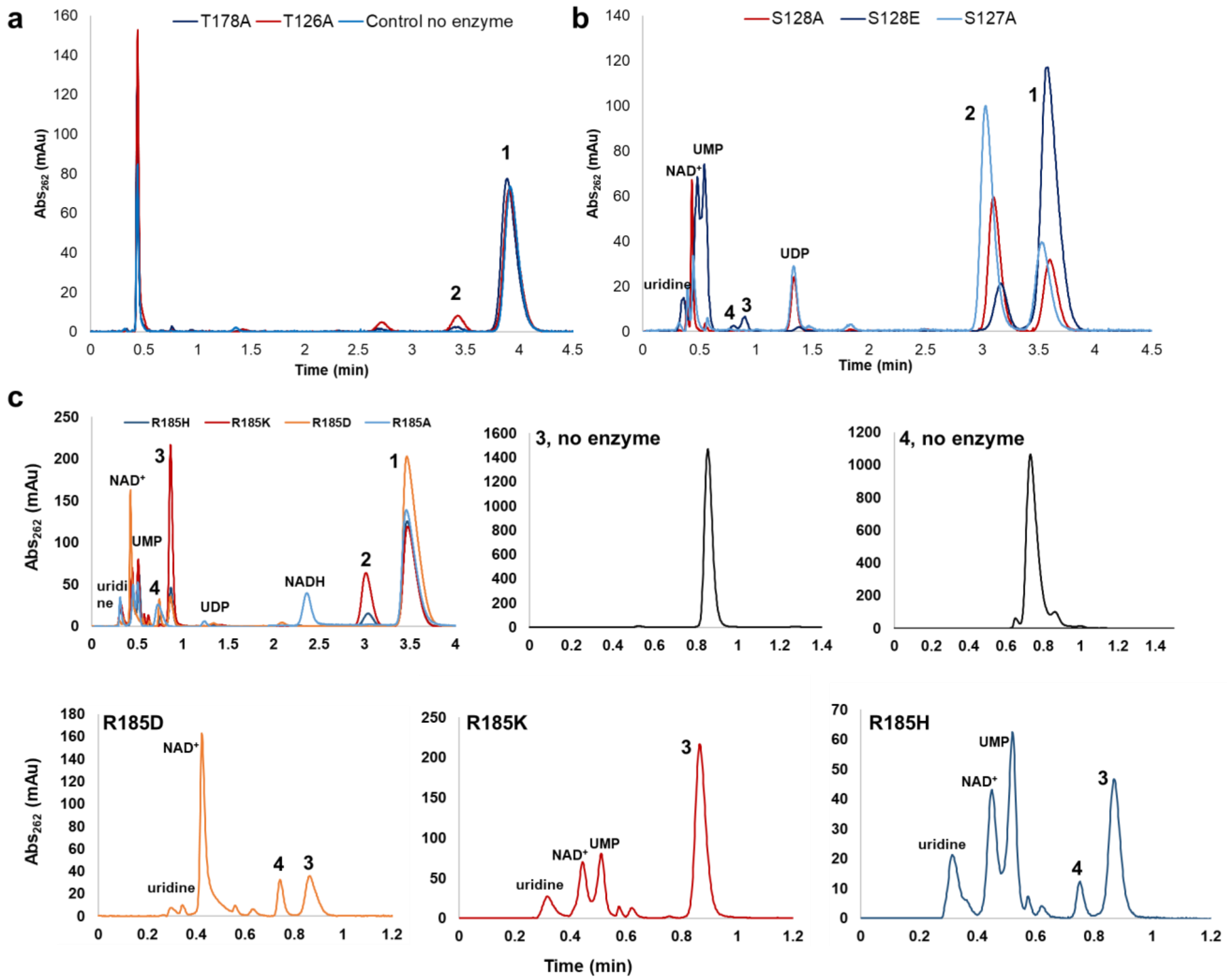

Figure S10. HPLC traces of the mixtures of BcUGAepi variants reacted with UDP-GlcA. **a**. Overlay of the HPLC chromatograms of T178A (dark blue), T126A (red) and control (without enzyme, light blue) reactions after 120 min reaction time. **b**. Overlay of the HPLC chromatograms of S128A (red), S128E (dark blue) and S127A (light blue) reactions after 120 min reaction time. The release of tiny amount of decarboxylated products UDP-Xyl (**3**) and UDP-4-keto-pentose (**4**) can be seen for the S128E reaction. **c**. HPLC chromatograms of R185 variants' reactions after 150 min reaction time overlaid (top left) and separately with a zoom-in into the region of the decarboxylated products (bottom). R185H (dark blue), R185K (red), R185D (orange) and R185A (light blue). The formation of UDP-GalA (**2**) in addition to the decarboxylated products **3** and **4** is only observed with R185K and R185H variants. HPLC chromatograms of the standards of isolated **3** and **4** (black) are shown to confirm the retention times.[24]

Discussion: The release of NADH in the R185A reaction (Figure S10c) is attributed to the presence of UDP-4-keto-pentose as the only reaction product. Additionally, the R185A enzyme contains a small amount of NADH incorporated during the recombinant expression in *E. coli.* The release of uridine, UMP and UDP is due to decomposition of UDP-GlcA under the reaction conditions. The degradation occurs in the absence of an enzyme (Figure S12), while non-optimal binding from an epimerase variant can further facilitate the decomposition, depending on the variant as well as on the protein concentration used (Figure S13).

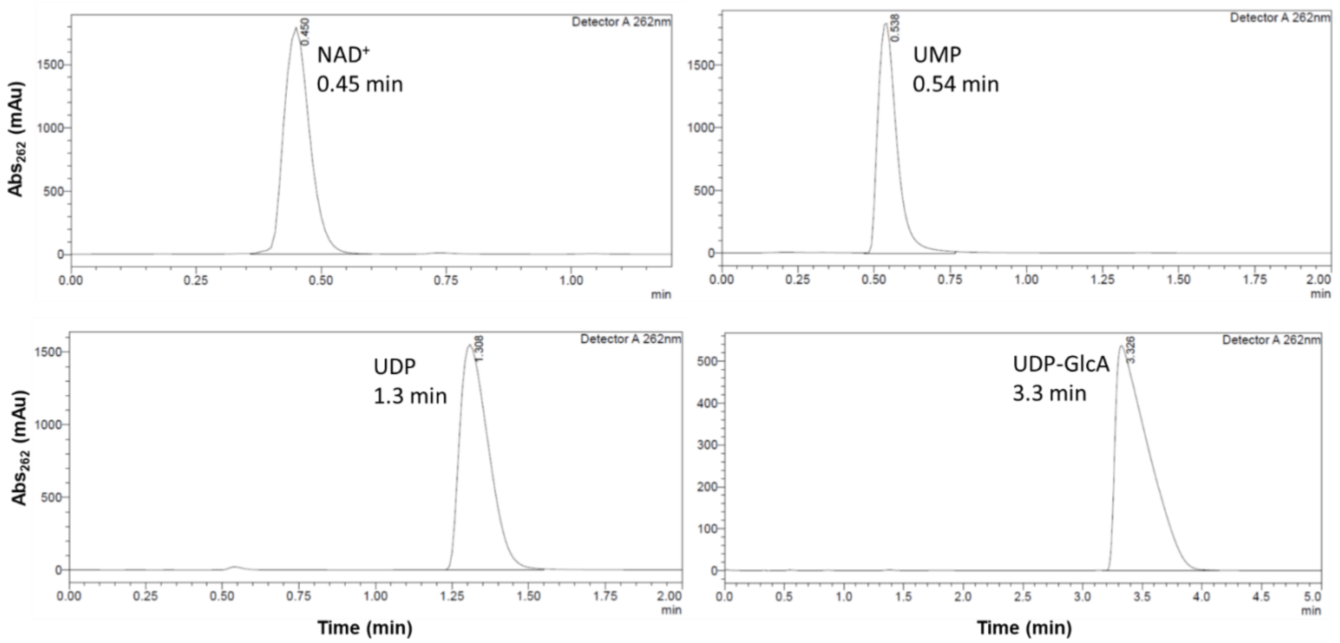

Figure S11. HPLC chromatograms of the standards of NAD^+, UMP, UDP and UDP-GlcA dissolved in distilled water.

Discussion: There are no decomposition products present when the individual standards are dissolved in water and measured on HPLC.

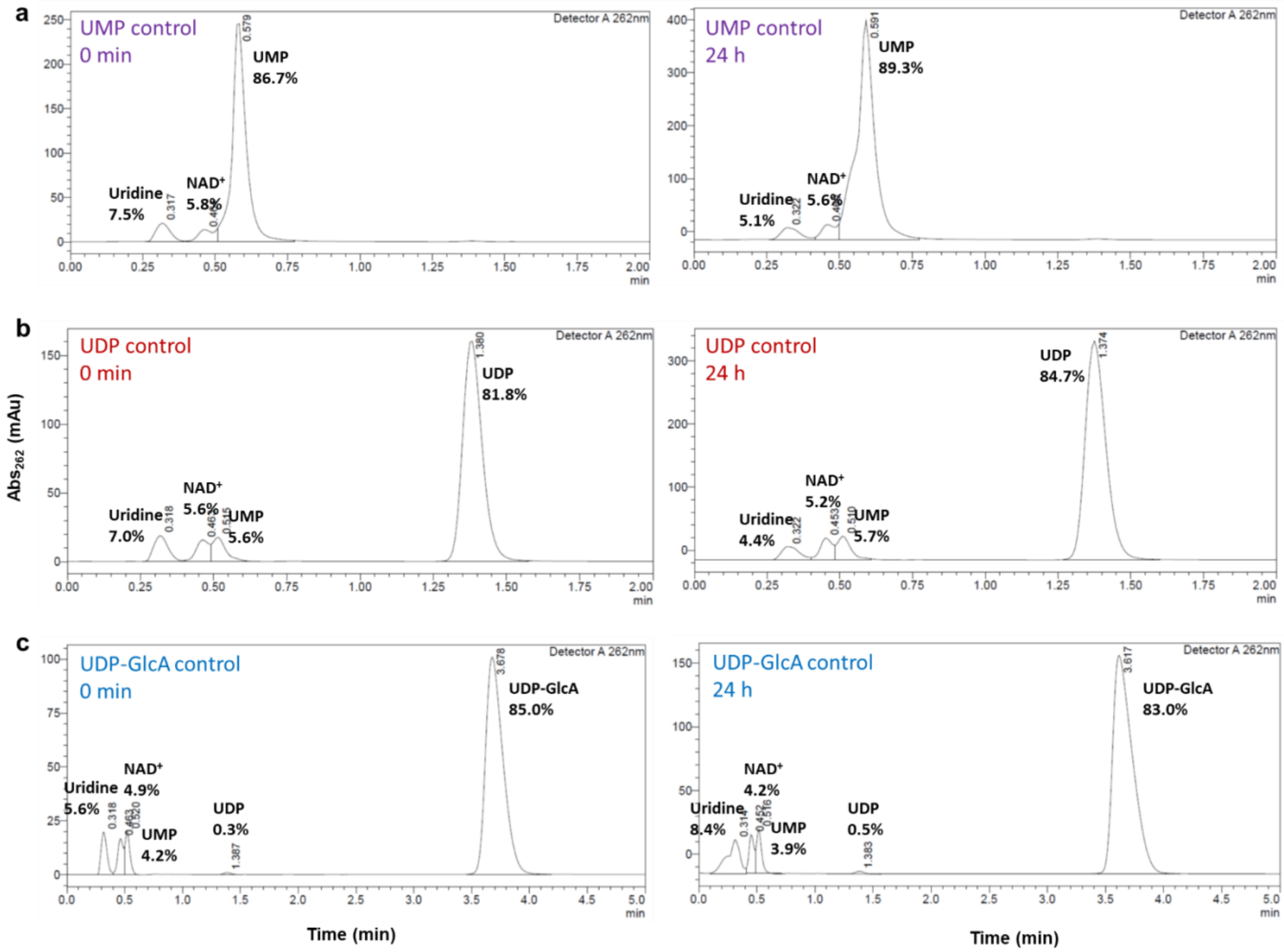

Figure S12. HPLC traces of the control reactions (without enzyme) performed for analyzing the stability of UMP (**a**), UDP (**b**) and UDP-GlcA (**c**) under the standard reaction conditions. The reactions were performed with 1 mM substrate (UMP/UDP/UDP-GlcA) and 100 µM NAD$^+$ in sodium phosphate buffer (50 mM Na_2HPO_4, 100 mM NaCl, pH 7.6) in final volume of 100 µL. The chromatograms at the start of the reaction (0 min) and after 24 h are displayed.

Discussion: The decomposition products (uridine from UMP; uridine and UMP from UDP; uridine, UMP and UDP from UDP-GlcA) are present already at the start of the reaction (performed without an enzyme). The amount of the decomposition products is not significantly changing within 24 h of incubation.

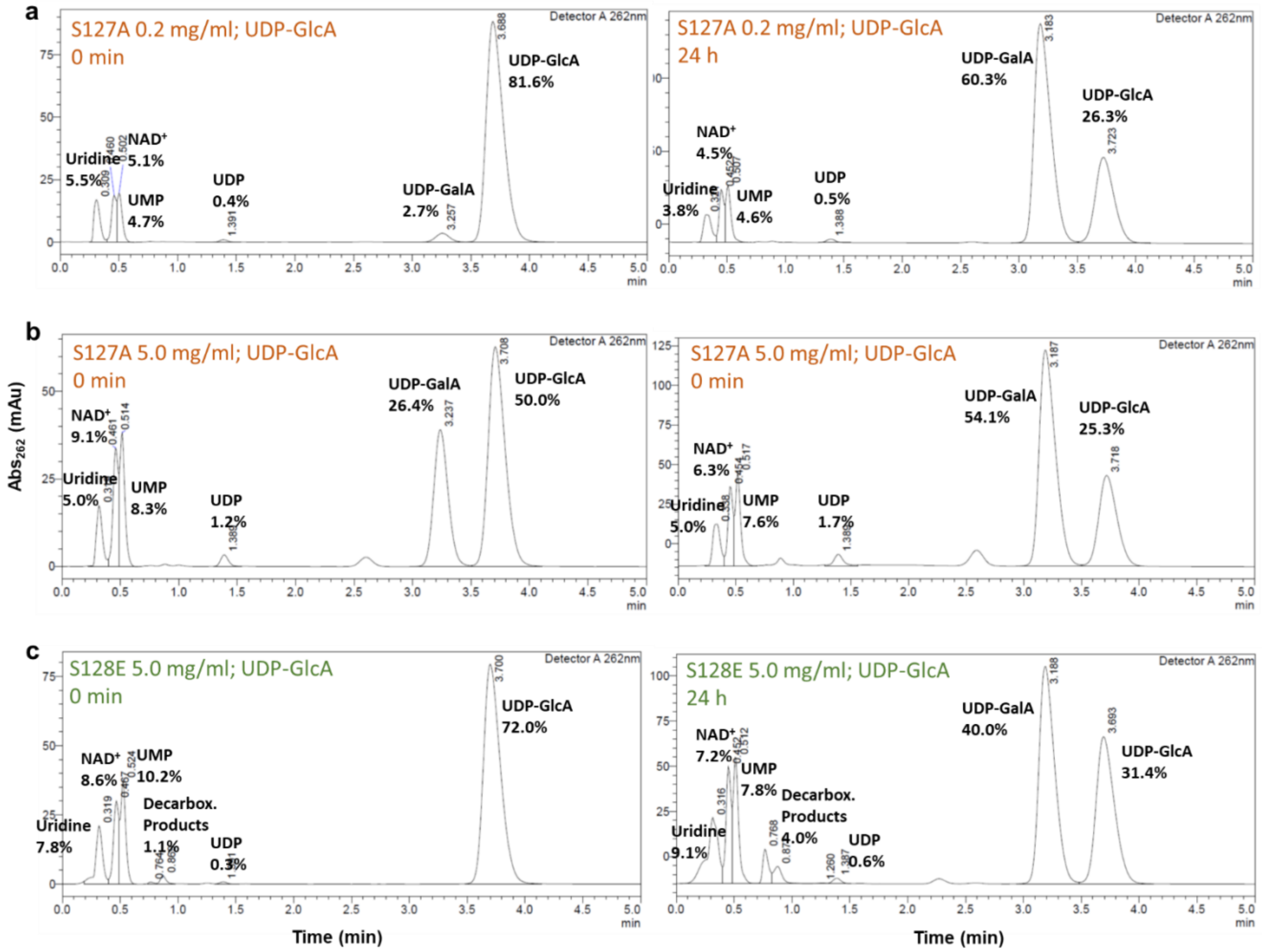

Figure S13. HPLC traces of the control reactions performed for analyzing the stability of UDP-GlcA in presence of epimerase variants S127A and S128E with different enzyme concentrations (0.2 mgmL^{-1}, 5.0 mgmL^{-1}). The reactions were carried out with 1 mM UDP-GlcA and 100 µM NAD$^+$ in sodium phosphate buffer (50 mM Na_2HPO_4, 100 mM NaCl, pH 7.6) in final volume of 100 µL. The chromatograms at the start of the reaction (0 min) and after 24 h are shown. **a**. Reaction of S127A variant with UDP-GlcA at low enzyme concentration (0.2 mgmL^{-1}). **b**. Reaction of S127A variant with UDP-GlcA at high enzyme concentration (5.0 mgmL^{-1}). **c**. Reaction of S128E enzyme (5.0 mgmL^{-1}) with UDP-GlcA.

Discussion: The comparison of S127A reactions with low (Figure S13a) and high (Figure S13b) enzyme concentration shows that the amount of decomposition products (uridine, UMP, UDP) slightly increases with increasing enzyme concentration. The degradation products are released immediately at the start of the reaction and the amount is not significantly changing over time, similarly to the control reactions without an enzyme (Figure S12). The effect of different enzyme variants on the amount of decomposition products was studied by comparing the reactions of S127A (Figure S13b) and S128E (Figure S13c) at the same enzyme concentration (5.0 mgmL^{-1}). The quantity of released uridine and UMP was marginally higher with S128E variant, while the amount of UDP was slightly more with the S127A enzyme. As a conclusion, the amount of the degradation products is dependent on both the enzyme variant and the enzyme concentration used, as can be seen in Figures S10 and S13. We consider the effect to be due to the composition of the reaction buffer and the enzyme solution used, rather than to an active input from the enzyme. The quantity of the decomposition products remains nearly unchanged over time.

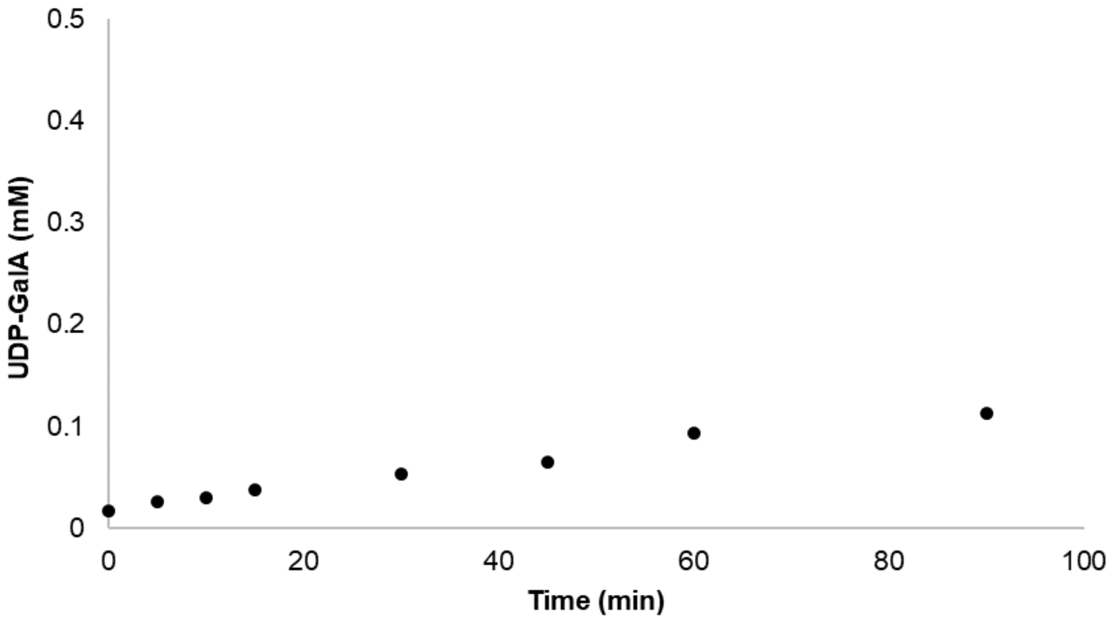

Figure S14. Time course of BcUGAepi_T126A catalyzed reaction with UDP-GlcA as a substrate. The reaction was performed with 1 mM UDP-GlcA, 100 µM NAD^+ and 270 µM (10 $mgmL^{-1}$) purified recombinant BcUGAepi_T126A in sodium phosphate buffer (50 mM Na_2HPO_4, 100 mM NaCl, pH 7.6) in final volume of 250 µL. The activity of T126A variant (0.08 $mUmg^{-1}$) was calculated from the linear part of the time course.

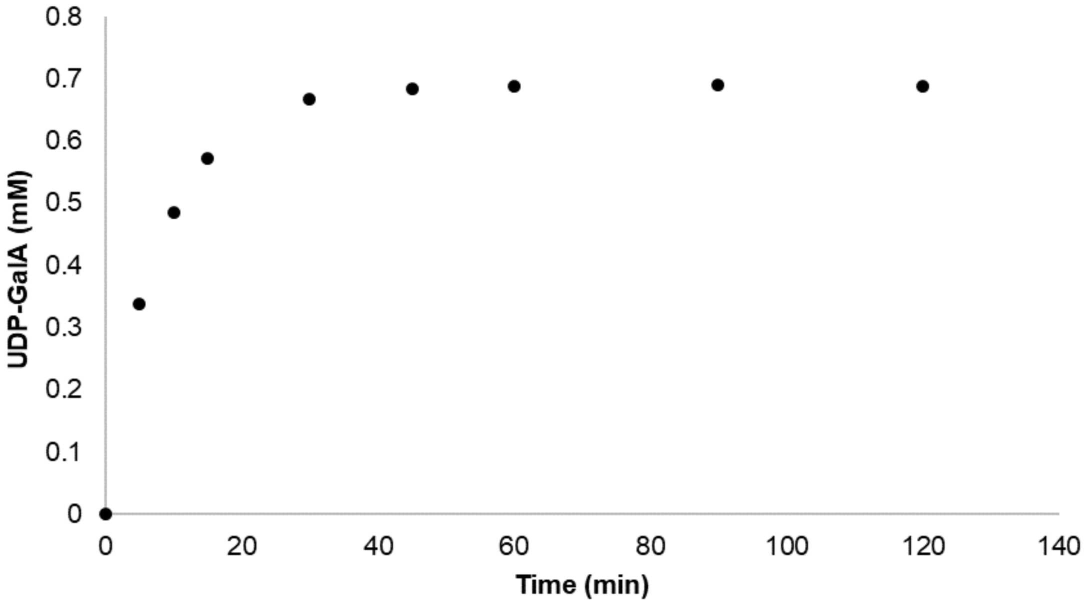

Figure S15. Time course of BcUGAepi_S127A catalyzed reaction with UDP-GlcA as a substrate. The reaction was performed with 1 mM UDP-GlcA, 100 µM NAD^+ and 5.4 µM (0.2 $mgmL^{-1}$) purified recombinant BcUGAepi_S127A in sodium phosphate buffer (50 mM Na_2HPO_4, 100 mM NaCl, pH 7.6) in final volume of 250 µL. The activity of S127A variant (24.3 $mUmg^{-1}$) was calculated from the initial velocity (linear part) of the time course.

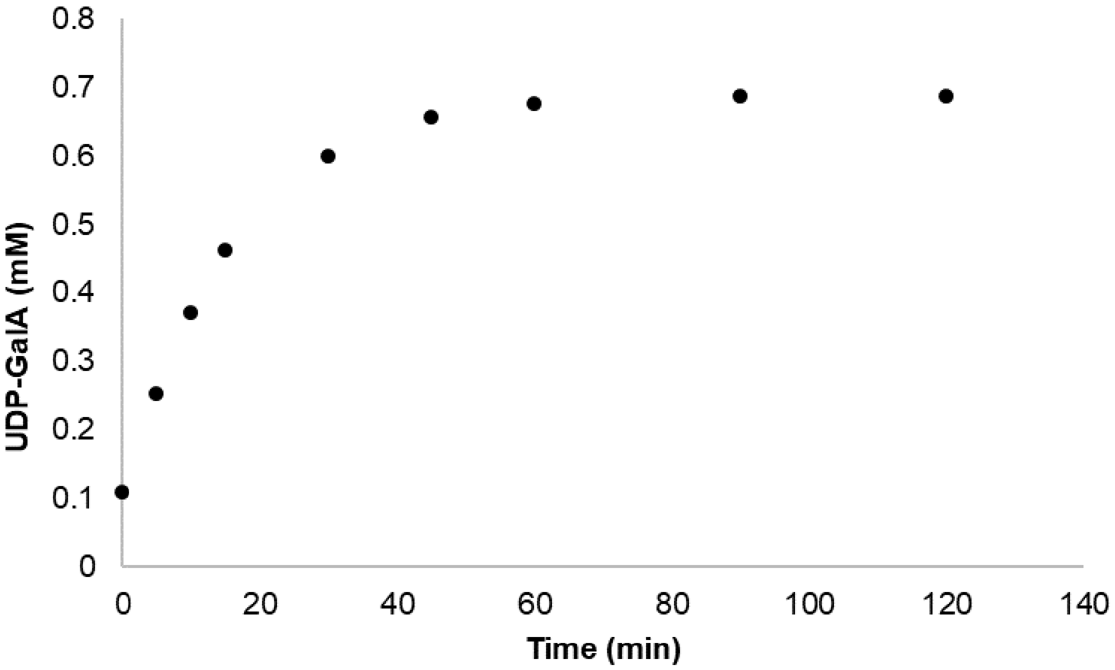

Figure S16. Time course of BcUGAepi_S128A catalyzed reaction with UDP-GlcA as a substrate. The reaction was performed with 1 mM UDP-GlcA, 100 µM NAD^+ and 5.4 µM (0.2 $mgmL^{-1}$) purified recombinant BcUGAepi_S128A in sodium phosphate buffer (50 mM Na_2HPO_4, 100 mM NaCl, pH 7.6) in final volume of 250 µL. The activity of S128A variant (11.8 $mUmg^{-1}$) was calculated from the initial velocity (linear part) of the time course.

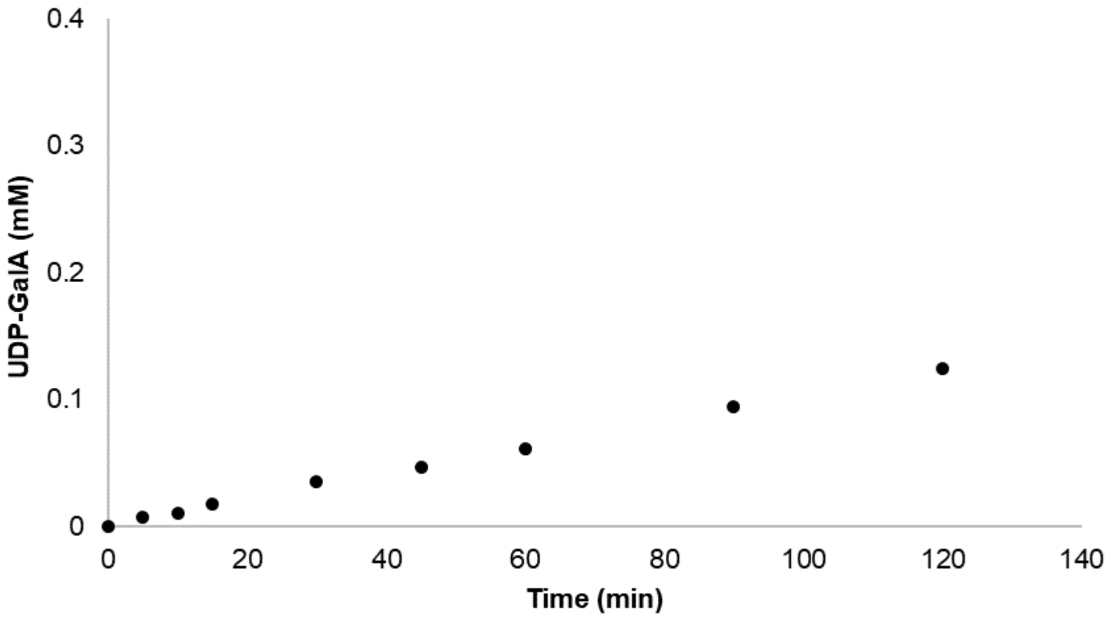

Figure S17. Time course of BcUGAepi_S128E catalyzed reaction with UDP-GlcA as a substrate. The reaction was performed with 1 mM UDP-GlcA, 100 µM NAD^+ and 135 µM (5 $mgmL^{-1}$) purified recombinant BcUGAepi_S128E in sodium phosphate buffer (50 mM Na_2HPO_4, 100 mM NaCl, pH 7.6) in final volume of 250 µL. The activity of S128E variant (0.2 $mUmg^{-1}$) was calculated from the time course.

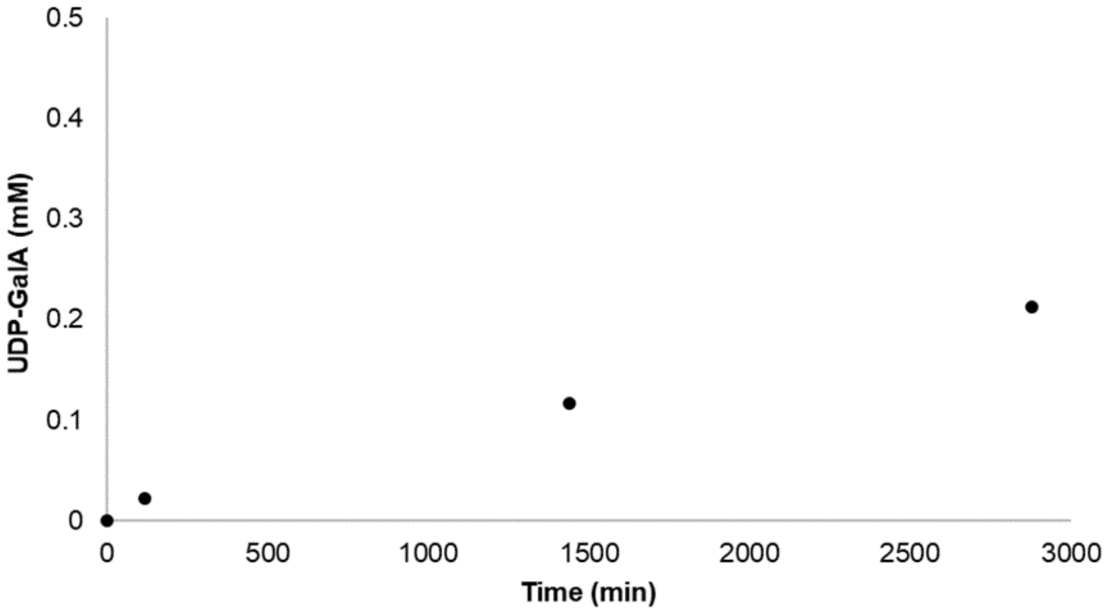

Figure S18. A brief time course of BcUGAepi_T178A catalyzed reaction with UDP-GlcA as a substrate. The reaction was performed with 1 mM UDP-GlcA, 100 µM NAD^+ and 43 µM (1.6 $mgmL^{-1}$) purified recombinant BcUGAepi_T178A in sodium phosphate buffer (50 mM Na_2HPO_4, 100 mM NaCl, pH 7.6) in final volume of 250 µL. The activity of T178A variant (0.09 $mUmg^{-1}$) was calculated from the time course.

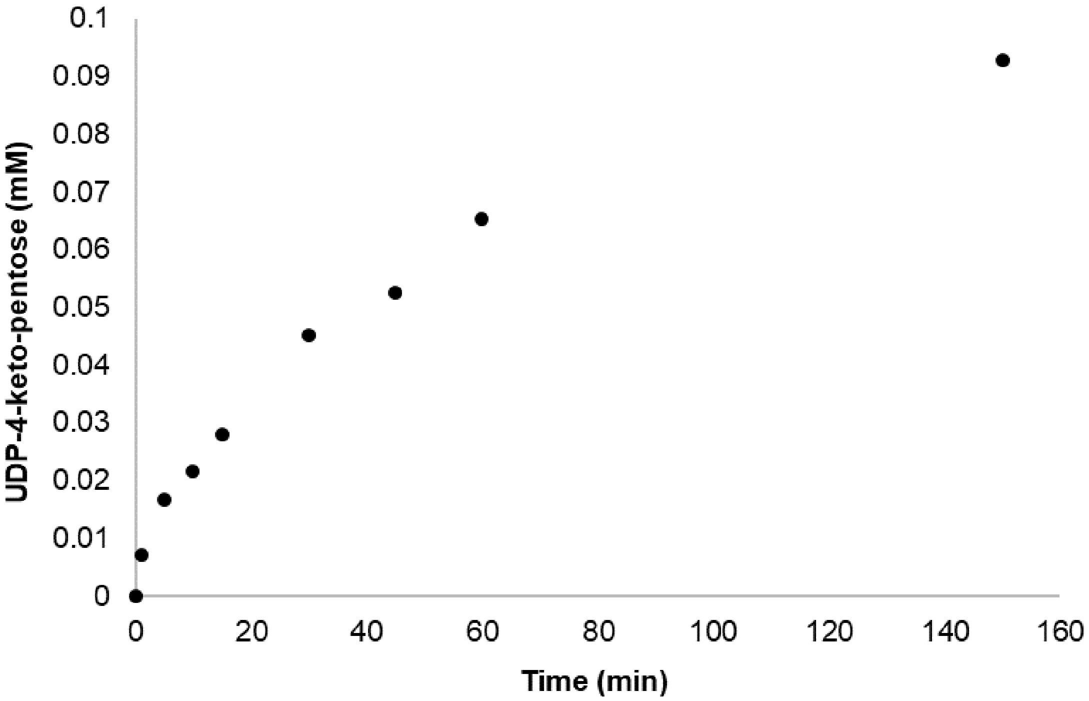

Figure S19. Time course of BcUGAepi_R185A catalyzed reaction with UDP-GlcA as a substrate. The reaction was performed with 1 mM UDP-GlcA, 100 µM NAD^+ and 270 µM (10 $mgmL^{-1}$) purified recombinant BcUGAepi_R185A in sodium phosphate buffer (50 mM Na_2HPO_4, 100 mM NaCl, pH 7.6) in final volume of 250 µL. The activity of R185A variant (0.05 $mUmg^{-1}$) was calculated from the linear part (excluding 0- and 1-min time points) of the time course.

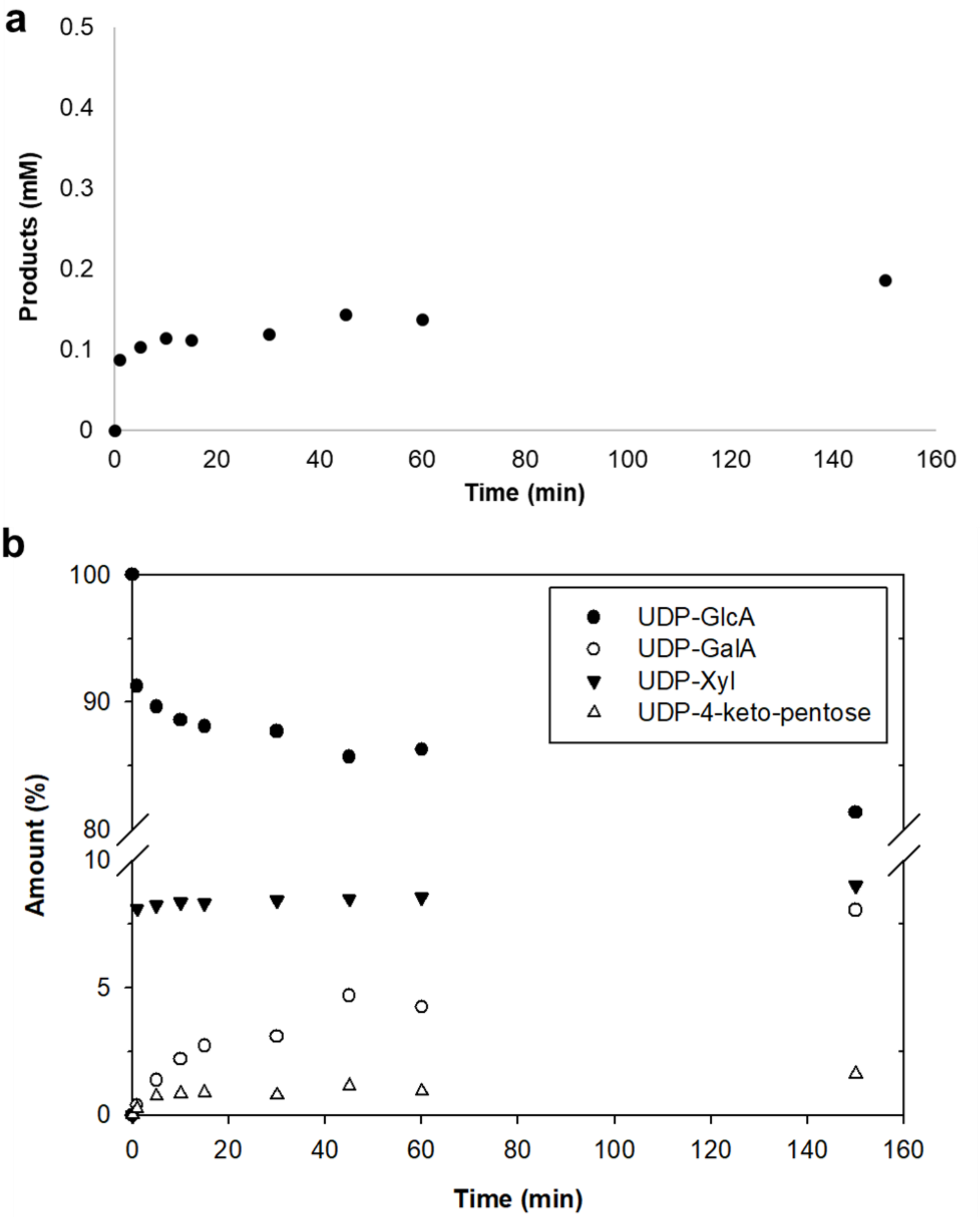

Figure S20. Time courses of BcUGAepi_R185H catalyzed reaction with UDP-GlcA as a substrate. **a**. Time course for the total amount of products (UDP-GalA, UDP-xylose, UDP-4-keto-pentose) used for the activity calculation. The activity of R185H variant (0.05 $mUmg^{-1}$) was calculated from the linear part (excluding 0- and 1-min time points) of the time course. **b**. Time course for showing the substrate and each product individually. The reaction was performed with 1 mM UDP-GlcA, 100 µM NAD^+ and 405 µM (15 $mgmL^{-1}$) purified recombinant BcUGAepi_R185H in sodium phosphate buffer (50 mM Na_2HPO_4, 100 mM NaCl, pH 7.6) in final volume of 250 µL.

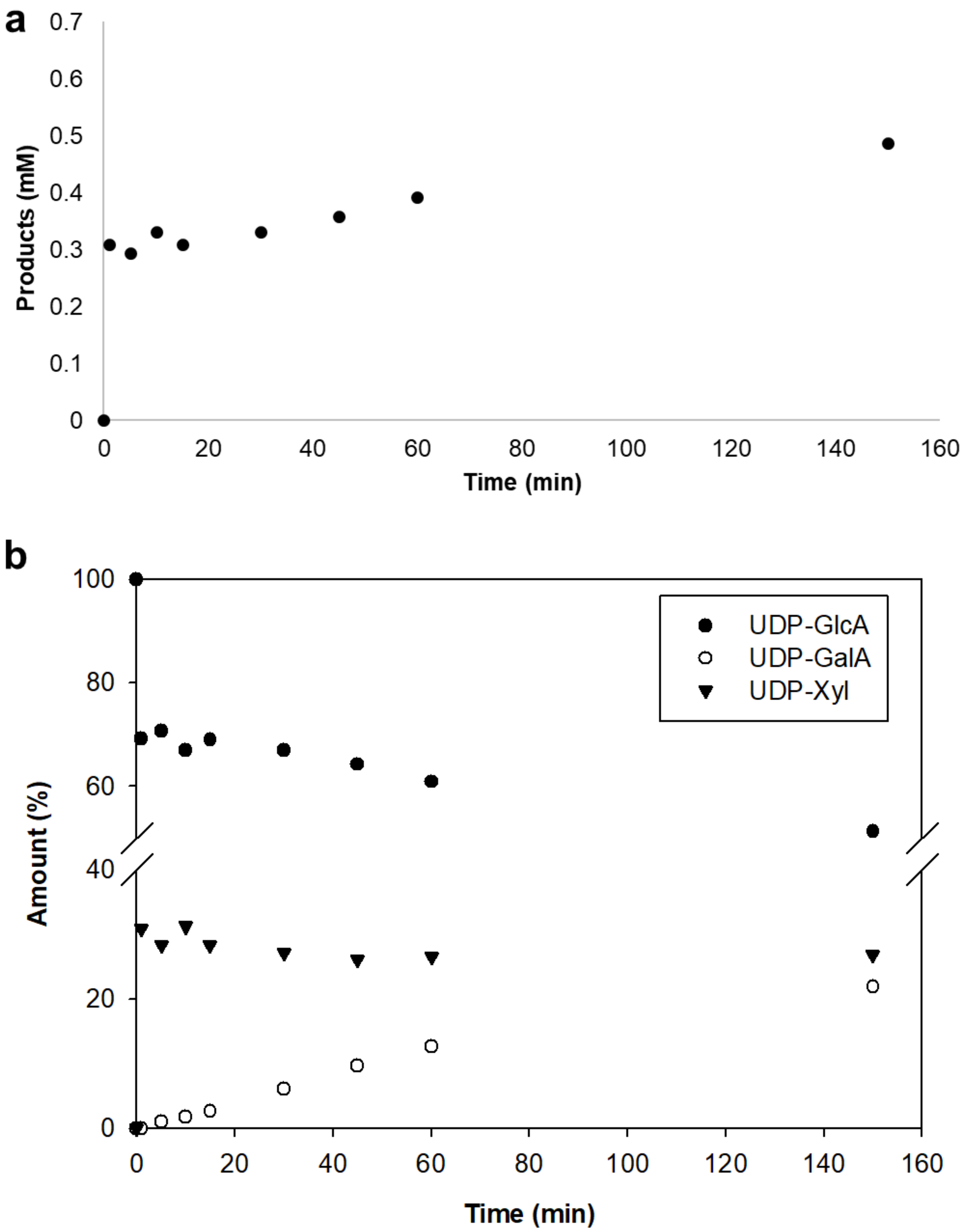

Figure S21. Time courses of BcUGAepi_R185K catalyzed reaction with UDP-GlcA as a substrate. **a**. Time course for the total amount of products (UDP-GalA, UDP-xylose, UDP-4-keto-pentose) used for the activity calculation. The activity of R185K variant (0.3 $mUmg^{-1}$) was calculated from the linear part (excluding 0- and 1-min time points) of the time course. **b**. Time course for showing the substrate and each product individually, UDP-4-keto-pentose was omitted for clarity since the amount stayed < 0.2% throughout the reaction. The reaction was performed with 1 mM UDP-GlcA, 100 µM NAD^+ and 127 µM (4.7 $mgmL^{-1}$) purified recombinant BcUGAepi_R185K in sodium phosphate buffer (50 mM Na_2HPO_4, 100 mM NaCl, pH 7.6) in final volume of 250 µL.

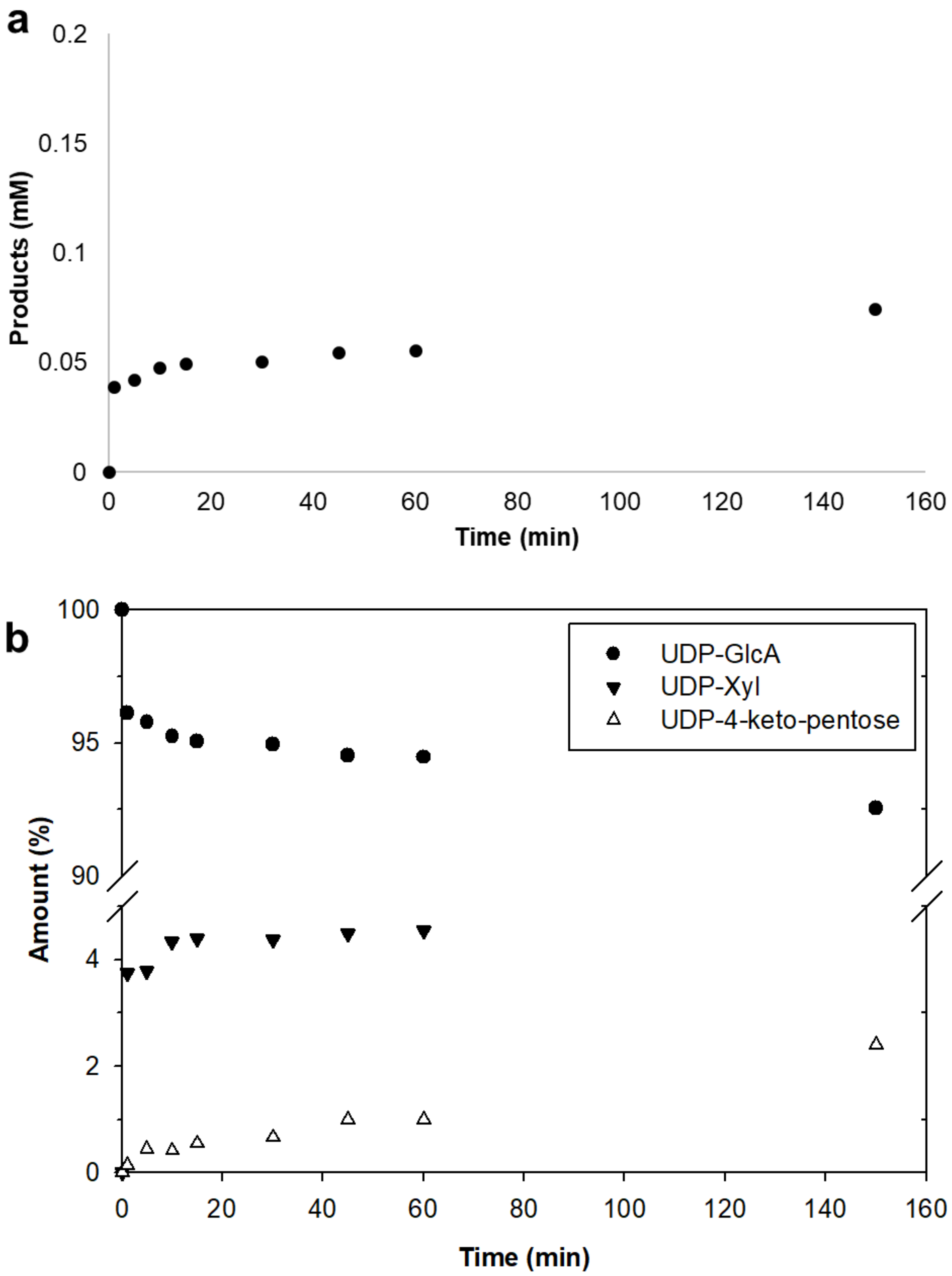

Figure S22. Time courses of BcUGAepi_R185D catalyzed reaction with UDP-GlcA as a substrate. **a**. Time course for the total amount of products (UDP-xylose, UDP-4-keto-pentose) used for the activity calculation. The activity of R185D variant (0.05 mUmg^{-1}) was calculated from the linear part (excluding 0- and 1-min time points) of the time course. **b**. Time course for showing the substrate and each product individually. The reaction was performed with 1 mM UDP-GlcA, 100 µM NAD^{+} and 405 µM (15 mgmL^{-1}) purified recombinant BcUGAepi_R185D in sodium phosphate buffer (50 mM Na_2HPO_4, 100 mM NaCl, pH 7.6) in final volume of 250 µL.

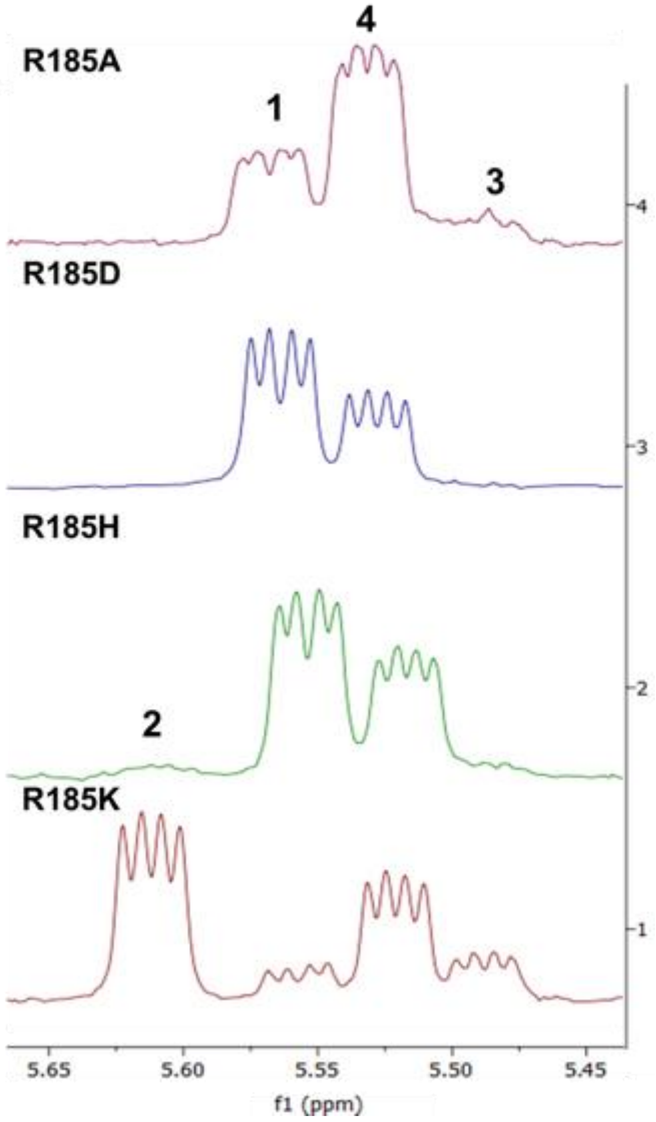

Figure S23. ^{1}H-NMR spectra of the reaction mixtures of R185 variants. Prior to NMR analysis, each reaction mixture was treated with ArnA to convert ~50% of the remaining substrate to UDP-4-keto-pentose to improve the resolution on the anomeric region (SI methods). The reactions were performed in H_2O, the enzymes removed by filtration and the remaining supernatant lyophilized prior to re-dissolving the compounds in D_2O. A close-up of the anomeric region is shown. UDP-glucuronic acid, **1**, 5.48 ppm; UDP-galacturonic acid, **2**, 5.52 ppm; UDP-xylose, **3**, 5.62 ppm; UDP-4-keto-pentose, **4**, 5.56 ppm. If UDP-arabinose was present, a signal would be seen at 5.65 ppm.[28]

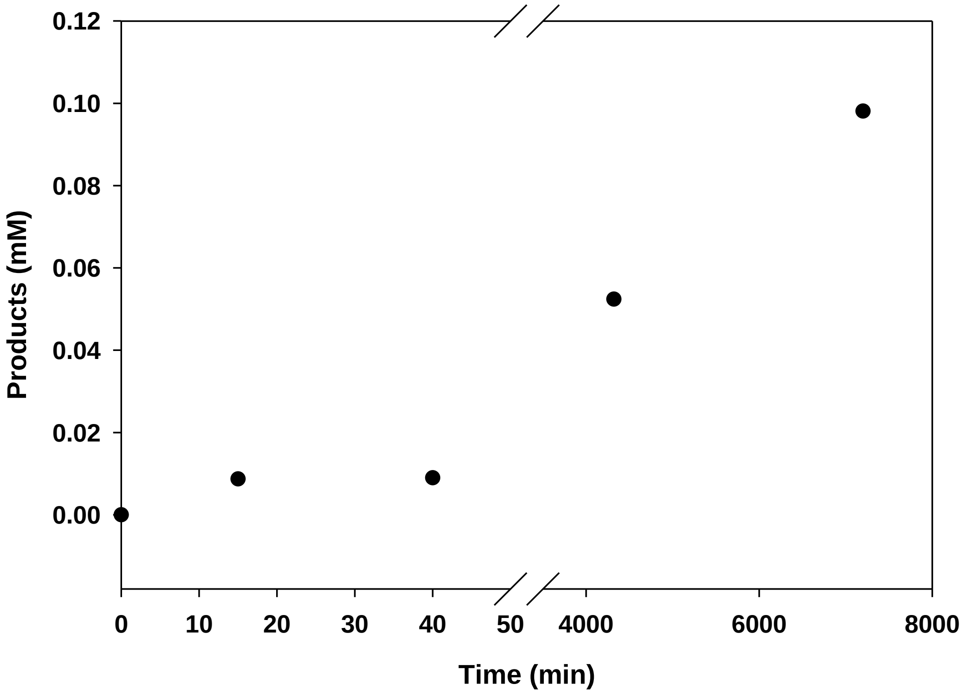

Figure S24. Time course of BcUGAepi_R185H (0.15 mgmL^{-1}, 4.05 µM) catalyzed reaction with UDP-GlcA (10 mM) to resolve the kinetics of the initial product burst. The reaction was supplemented with 1 mM NAD$^+$ and performed in phosphate buffer (50 mM Na_2HPO_4, 100 mM NaCl, pH 7.6) in final volume of 100 µL. The burst activity of R185H variant (4.0 mUmg^{-1}) was calculated from the first two time points (0, 15 min) and the after-burst activity (0.06 mUmg^{-1}) from the time points 40-7200 min. The y-axis corresponds to the total amount of products UDP-xylose and UDP-4-keto-pentose.

Discussion: The calculated burst activity (4.0 mUmg^{-1}), is in agreement with the value estimated from the time course with high enzyme concentration (~5 mUmg^{-1}; Figure S20; main text Table 1). The activity after the burst (0.06 mUmg^{-1}) correlates well with the value (0.05 mUmg^{-1}) obtained from the high enzyme concentration reaction (Figure S20, main text Table 1).

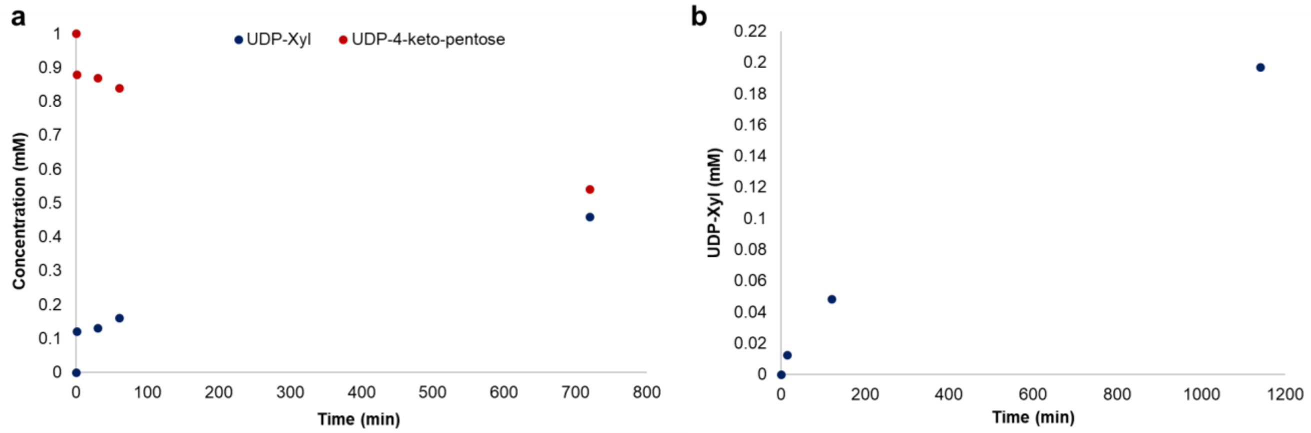

Figure S25. Time courses of BcUGAepi wild type catalyzed reactions with UDP-4-keto-pentose as a substrate. The reactions were performed with 1 mM (**a**) or 10 mM (**b**) UDP-4-keto-pentose and 10 mM NADH in sodium phosphate buffer (50 mM Na_2HPO_4, 100 mM NaCl, pH 7.6) in final volume of 250 µL. **a**. Time course with high enzyme concentration (8 mgmL^{-1}; 216 µM) without additional UDP-xylose. **b**. Time course with decreased enzyme concentration (1 mgmL^{-1}; 27 µM) where UDP-xylose (100 µM) was supplemented at the start of the reaction.

Discussion: Due to the low steady-state activity of the R185 variants, the experiments were performed with the wild-type enzyme. Reduction of UDP-4-keto-pentose by enzyme-NADH (see Scheme 1 of main text) was analyzed. When offered excess NADH (here 10 mM) in addition to UDP-4-keto-pentose, conversion into UDP-Xyl was observed, as shown in panel a of this figure. The reaction involves a significant UDP-Xyl burst that amounts to 50% of the enzyme molarity. The product burst activity (1 min) is 15 mUmg^{-1} and the activity after the burst (1-720 min) is 0.06 mU/mg (Figure S25a). Preincubation of the enzyme with NADH did not change the observed rates and the overall kinetic behavior. The possibility that the exchange of NAD^+ by NADH is rate-determining for the overall enzymatic reduction is thus excluded. Decreasing the enzyme concentration 8-fold to 1 mgmL-1 (27 µM) and increasing the substrate concentration 10-fold (10 mM) were not helpful for eliminating the fast product release (data not shown), as also observed with the R185H variant reacted with UDP-GlcA (Figure S24). Increasing the substrate concentration to 10 mM and decreasing the enzyme concentration to 1 mgmL-1 (27 µM) while supplementing the reaction with UDP-xylose (100 µM, added prior to the enzyme) eliminated the product burst (Figure S25b), as expected from Scheme 1 of main text. The initial activity of 0.8 mUmg^{-1} was calculated from the time points 0-15 min and activity of 0.1 mUmg^{-1} from the samples 120-1140 min (Figure S25b), demonstrating a usual kinetic behavior.

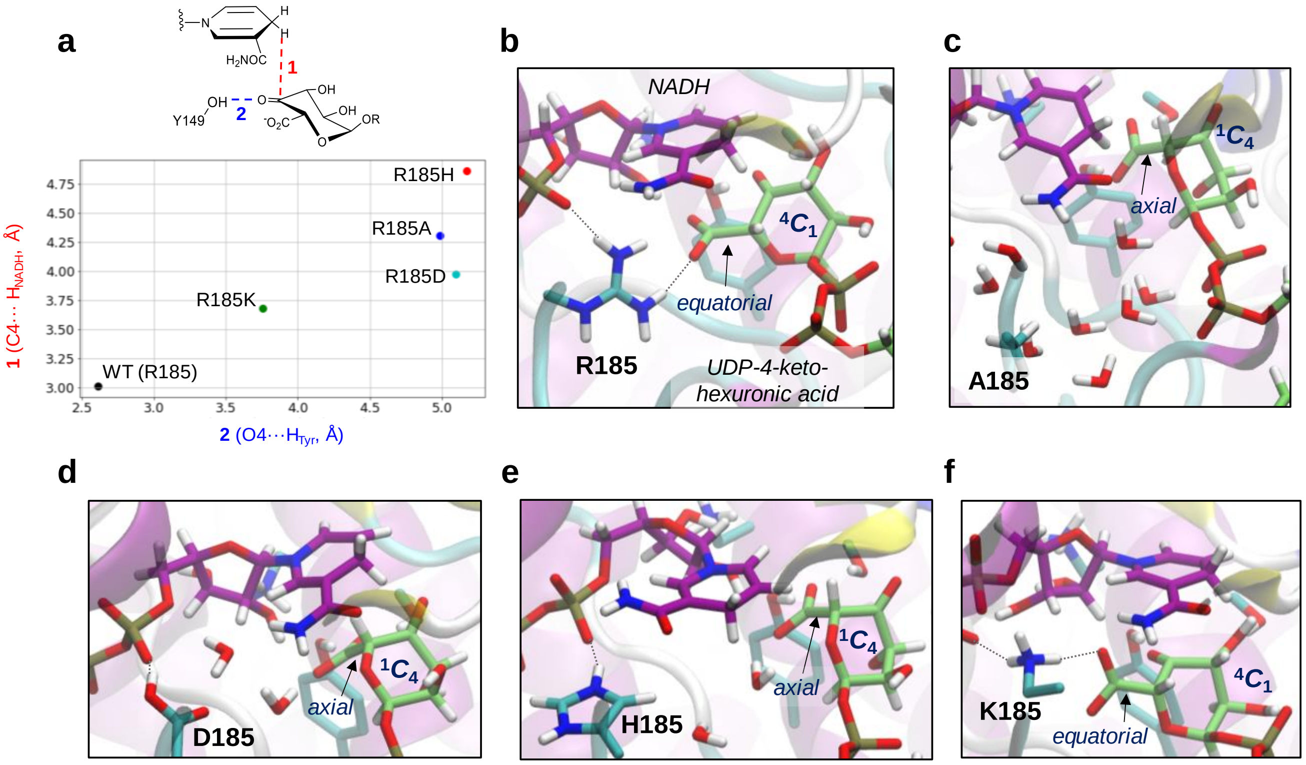

Figure S26. a. 2D representation of the values of the catalytic distances (O4···H_{Tyr} and C4···H_{NADH}) of the wild type enzyme and R185 variants obtained from MD simulations of the 4-keto-hexuronic acid intermediate after rotation in the active site of BcUGAE (**I**^{ROT} state). Every point corresponds to the average value over 200 ns of MD simulations. **b-f**. Representative structures of the active site configuration for the wild type and R185 variants.

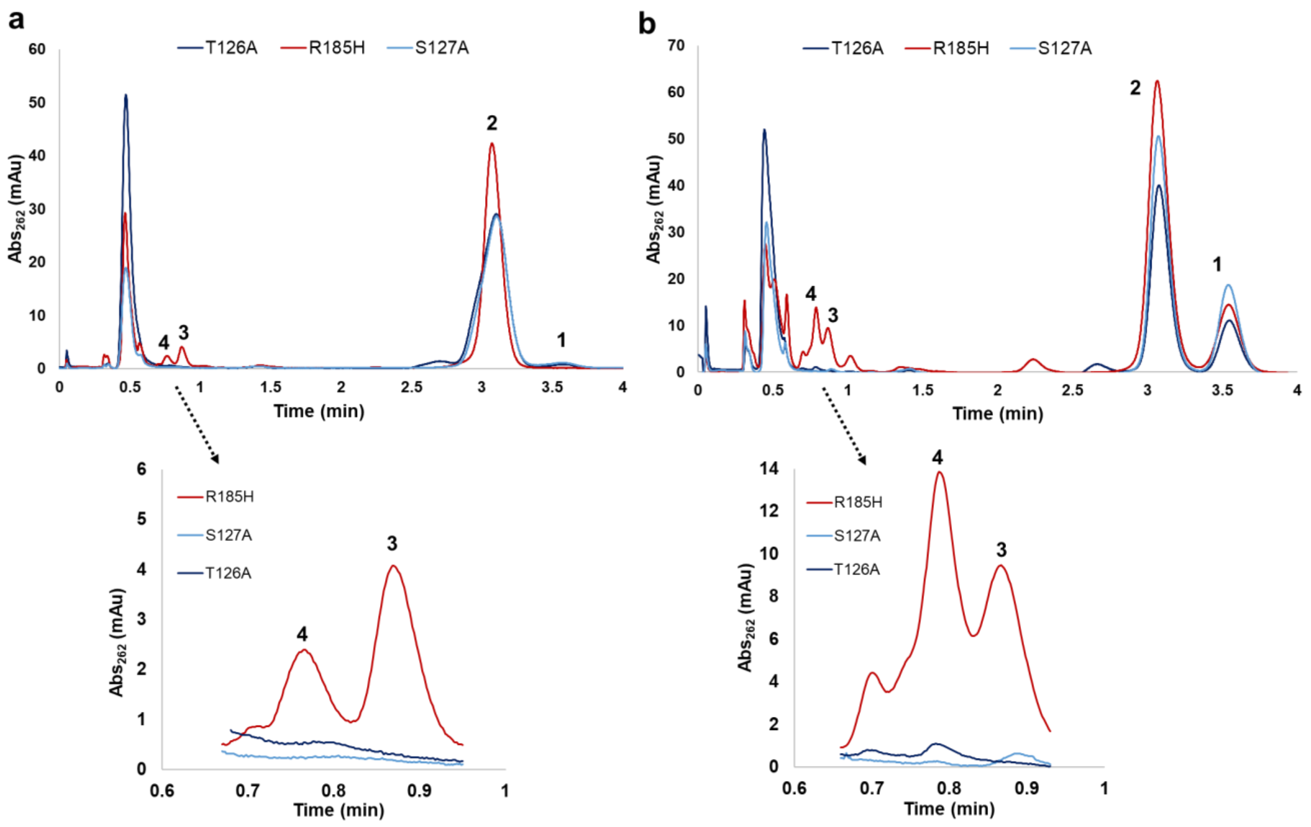

Figure S27. HPLC chromatograms from the reactions of BcUGAepi variants T126A (dark blue), S127A (light blue) and R185H (red) reacted with UDP-galacturonic acid (**2**). **a**. Overlay of the chromatograms after 1 min of reaction. **b**. Overlay of the chromatograms after 16 h reaction time. Zoom-in into the region of 0.6-1 min shows a trace amount of decarboxylated products in the T126A (UDP-4-keto-pentose, **4**) and S127A (UDP-xylose/UDP-arabinose, **3**) reactions after 16 h. UDP-glucuronic acid, **1**; UDP-galacturonic acid, **2**; UDP-xylose/UDP-L-arabinose, **3**; UDP-4-keto-pentose, **4**.

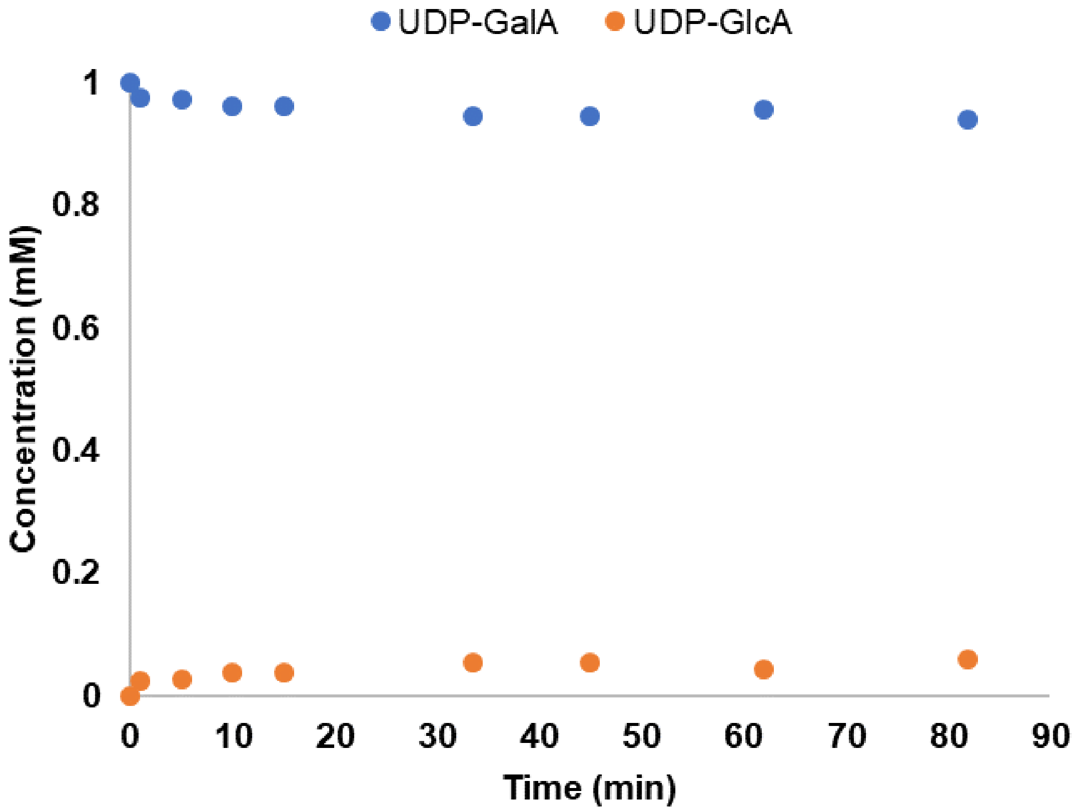

Figure S28. Time course of BcUGAepi_T126A catalyzed reaction with UDP-GalA as a substrate. The reaction was performed with 1 mM UDP-GalA, 100 µM NAD^+ and 270 µM (10 $mgmL^{-1}$) purified recombinant BcUGAepi_T126A in sodium phosphate buffer (50 mM Na_2HPO_4, 100 mM NaCl, pH 7.6) in final volume of 250 µL. The activity of T126A variant (0.09 $mUmg^{-1}$) was calculated from the linear part (excluding 0 and 1 min time points) of the time course.

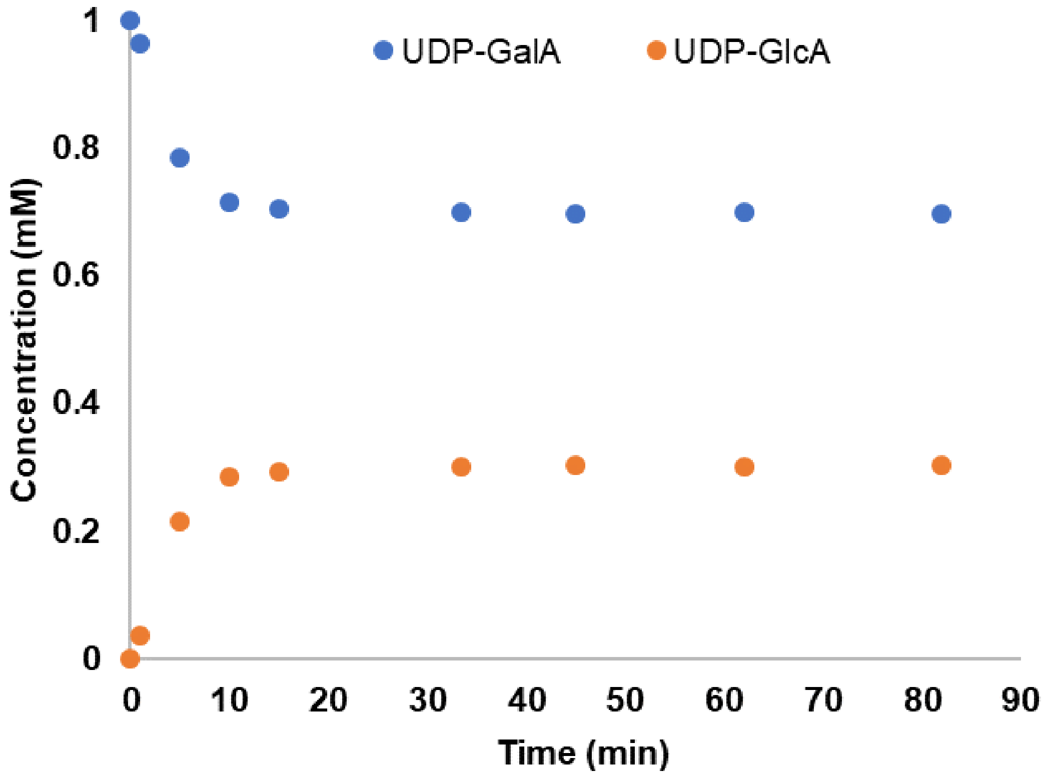

Figure S29. Time course of BcUGAepi_S127A catalyzed reaction with UDP-GalA as a substrate. The reaction was performed with 1 mM UDP-GalA, 100 µM NAD^+ and 5.4 µM (0.2 $mgmL^{-1}$) purified recombinant BcUGAepi_T126A in sodium phosphate buffer (50 mM Na_2HPO_4, 100 mM NaCl, pH 7.6) in final volume of 250 µL. The activity of S127A variant (218 $mUmg^{-1}$) was calculated from the linear part (0-5 min) of the time course.

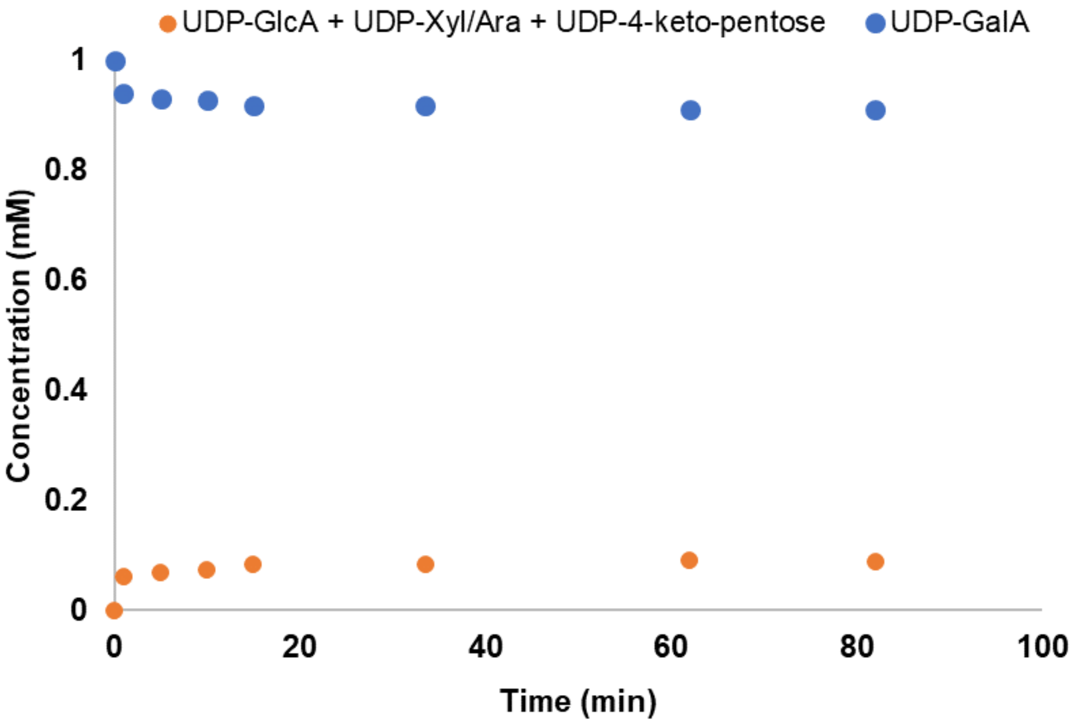

Figure S30. Time course of BcUGAepi_R185H catalyzed reaction with UDP-GalA as a substrate. The reaction was performed with 1 mM UDP-GalA, 100 µM NAD^+ and 405 µM (15 $mgmL^{-1}$) purified recombinant BcUGAepi_R185H in sodium phosphate buffer (50 mM Na_2HPO_4, 100 mM NaCl, pH 7.6) in final volume of 250 µL. The activity of R185H variant (0.1 $mUmg^{-1}$) was calculated from the time points 1-15 min.

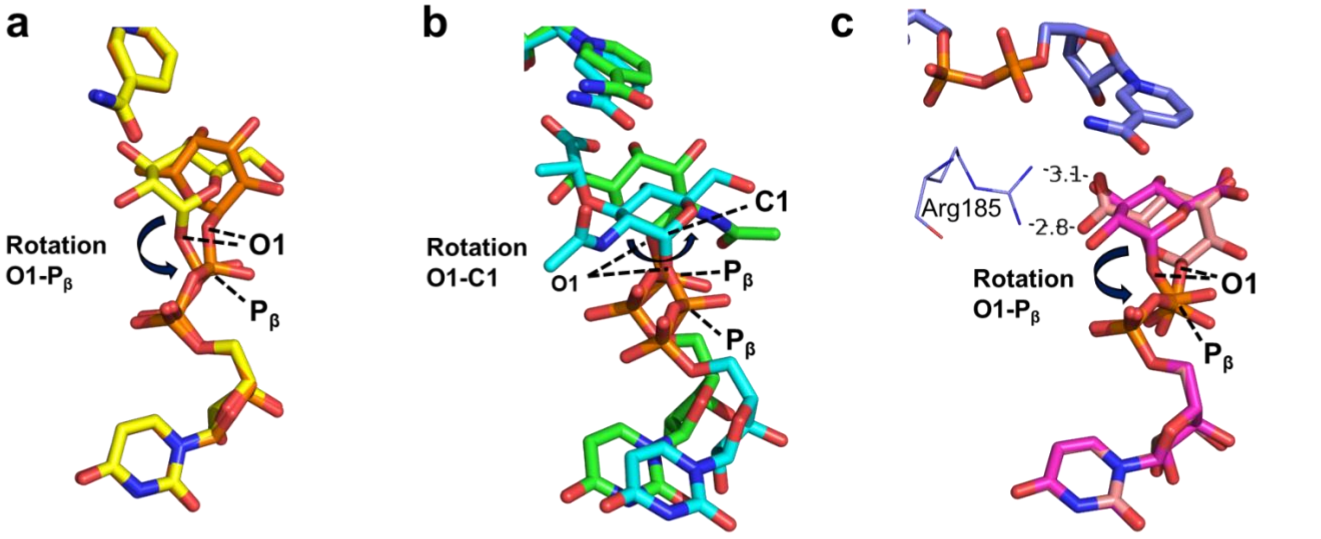

Figure S31. Overlaid active site close-ups of substrate and product complexes of GALE, UDP-GlcNAc 4-epimerase and UGAepi. **a.** Top-view on the overlay of *E. coli* GALE complexed with UDP-Glc (yellow carbons, PDB: 1XEL)[29] and UDP-Gal (orange, PDB: 1A9Z)[30] illustrating the rotational movement around the bond of β-phosphate (P_β) and O1. **b.** Structures of UDP-GlcNAc 4-epimerases MrWbpP in complex with a substrate analog UDP-MurNAc (cyan, PDB: 6DNT)[31] and PaWbpP complexed with UDP-GalNAc (green, PDB: 1SB8)[32] illustrating the rotational movement from the bond of anomeric carbon and O1. **c.** Top-view on the equilibrium complex of BcUGAepi (PDB: 6ZLK)[1] with UDP-GlcA (pink) and UDP-GalA (salmon) showing the rotation from the bond of β-phosphate and O1. The Arg185 residue important for binding the carboxylate moiety of UDP-GalA is highlighted with lines.

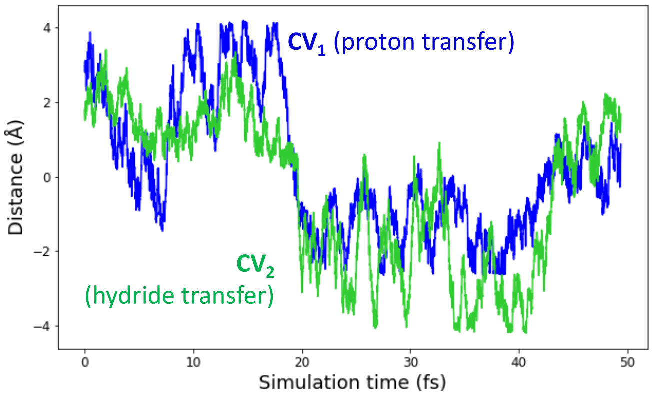

Figure S33. Evolution of the collective variables (CV_1 and CV_2) during the QM/MM metadynamics simulation of the oxidation reaction.

(a)

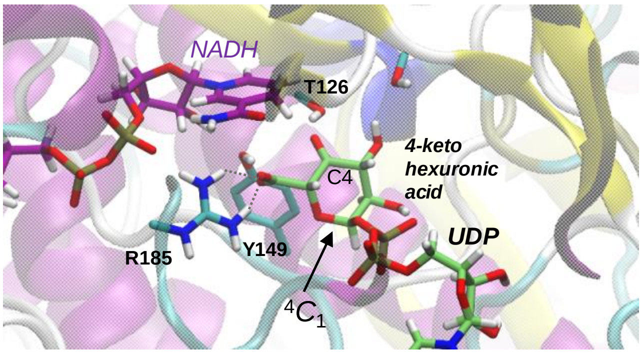

(b)

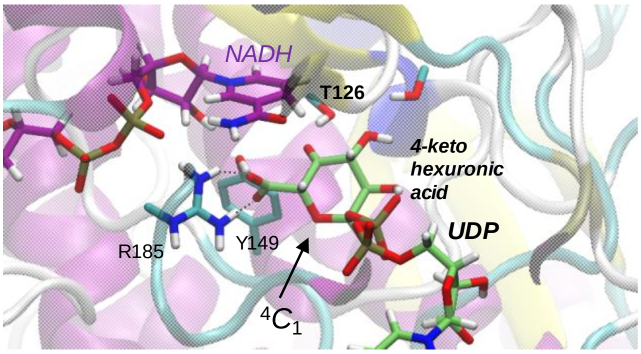

Figure S34. a. State $\mathbf{I}^{\mathbf{ROT}}$ obtained from the crystal structure of the enzyme complex with NAD^+ and UDP-GlcA (PDB entry 6ZLD), after QM/MM metadynamics simulations of the oxidation step and metadynamics simulations of 4-keto-hexuronic acid rotation in the active site. The picture corresponds to the energy minimized structure. **b**. State $\mathbf{I}^{\mathbf{ROT}}$ obtained from the crystal structure of the enzyme complex with NAD^+ and UDP-GalA (PDB entry 6ZLL), after manual conversion of GalA into 4-keto-hexuronic acid and suitable protonation of active site residues and the NADH cofactor. The picture corresponds to an energy minimized structure.

3. Supporting Tables

Table S1. Average values of the most relevant distances in the active site at the stationary states (minima and transition state TS) along the reaction coordinate.

Distance	MC'	MC	TS	I
C4' – H	2.64 ± 0.12	2.61 ± 0.14	1.38 ± 0.08	1.11 ± 0.03
C4 – H	1.12 ± 0.04	1.08 ± 0.03	1.39 ± 0.08	2.74 ± 0.38
O4 – H4	0.99 ± 0.02	0.98 ± 0.02	1.57 ± 0.11	1.99 ± 0.20
O_{Tyr} – H4	3.79 ± 0.32	1.74 ± 0.10	1.05 ± 0.04	1.00 ± 0.03

Table S2. Dihedral angle parameters obtained for 4-keto-hexuronic acid, according to the energy expression (Vn/2) [1 + cos(n φ – γ)].[6,33]

Angle	$V_n/2$	γ	n
Os-Cg-C-Cg	2.766	0.000	1
Os-Cg-C-Cg	0.527	180.000	2
Os-Cg-C-Cg	0.345	0.000	3
Cg-C-Cg-H1	0.192	180.000	1
Cg-C-Cg-H1	1.190	0.000	2
Cg-C-Cg-H1	0.896	0.000	3
Cg-C-Cg-Cg	0.405	0.000	1
Cg-C-Cg-Cg	2.120	0.000	2
Cg-C-Cg-Cg	0.361	0.000	3
Os-Cg-C-O	2.270	0.000	1
Os-Cg-C-O	2.254	0.000	2
Os-Cg-C-O	0.899	0.000	3
Cg-C-Cg-Oh	0.297	0.000	1
Cg-C-Cg-Oh	1.874	180.000	2
Cg-C-Cg-Oh	0.702	180.000	3
C-Cg-C-O2	0.342	180.000	1
C-Cg-C-O2	0.423	180.000	2
C-Cg-C-O2	0.529	180.000	3
Cg-C-Cg-C	3.286	180.000	1
Cg-C-Cg-C	0.103	180.000	2
Cg-C-Cg-C	0.371	180.000	3
O-C-Cg-C	0.506	0.000	1
O-C-Cg-C	0.701	0.000	2
O-C-Cg-C	0.775	0.000	3

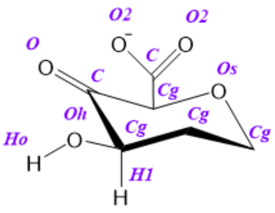

Table S3. Energy barriers obtained from several metadynamics simulations of sugar rotation starting either from the **I** or $\mathbf{I}^{ROT}$ states.

Replica	Free energy barrier $I \rightarrow I^{ROT}$	Replica	Free energy barrier $I \leftarrow I^{ROT}$
**1**	12.77	**7**	11.19
**2**	9.35	**8**	9.44
**3**	9.09	**9**	10.5
**4**	12.13	**10**	11.27
**5**	10.76	**12**	11.43
**6**	10.96	**13**	11.36
**Mean**	10.84		10.87
**StdDev**	1.46		0.77

4. Supporting Movie Caption

Trajectory of the metadynamics simulation corresponding to the rotation of the 4-keto-intermediate in the active site of BcUGAepi. The sidechain of the substrate and relevant active site residues are shown in licorice representation, whereas the rest of the enzyme is shown as cartoon backbone representation. Carbon atoms of the substrate are shown in green color while those of protein residues are in light blue.

5. References

[1] L. G. Iacovino, S. Savino, A. J. E. Borg, C. Binda, B. Nidetzky, A. Mattevi, *J. Biol. Chem.* **2020**, *295*, 12461–12473.
[2] M. H. M. Olsson, C. R. Søndergaard, M. Rostkowski, J. H. Jensen, *J. Chem. Theory Comput.* **2011**, *7*, 525–537.
[3] B. Gerratana, W. W. Cleland, P. A. Frey, *Biochemistry* **2001**, *40*, 9187–9195.
[4] Y. Wang, X. Li, J. Wei, X. Zhang, Y. Liu, *J. Chem. Inf. Model.* **2022**, *62*, 632–646.
[5] G. Ma, L. Dong, Y. Liu, *RSC Adv.* **2014**, *4*, 35449–35458.
[6] D. A. Case et al., AMBER 2018. University of California, San Francisco 2018.
[7] J. A. Maier, C. Martinez, K. Kasavajhala, L. Wickstrom, K. E. Hauser, C. Simmerling, *J. Chem. Theory Comput.* **2015**, *11*, 3696-3713.
[8] K. N. Kirschner, A. B. Yongye, S. M. Tschampel, J. Gonza, C. R. Daniels, B. Lachele Foley, R. J. Woods, *J. Comput. Chem.* **2008**, *29*, 622-655.
[9] W. L. Jorgensen, J. Chandrasekhar, J. D. Madura, *J. Chem. Phys* **1983**, *79*, 926-935.
[10] J. J. Pavelites, J. Gao, P. A. Bash, A. D. Mackerell, *J. Comput. Chem.* **1997**, *18*, 221-239.
[11] D. J. Frisch, M. J. Trucks, G. W. Schlegel, H. B. Scuseria et al. Gaussian 09, Revision B.01. Wallingford CT, 2009.
[12] J. P. Ryckaert, G. Ciccotti, H. J. C. Berendsen, *J. Comput. Phys.* **1977**, *23*, 327–341.
[13] A. Laio, J. Vandevondele, U. Rothlisberger, *J. Chem. Phys* **2002**, *116*, 6941-6947.
[14] R. Car, M. Parrinello, *Phys. Rev. Lett.* **1985,** *55*, 2471-2474.
[15] CPMD Program, Copyright IBM Corp. 1990-2003, Copyright MPI für Festkörperforschung, Stuttgart 1997-2001.
[16] W. Humphrey, A. Dalke, K. Schulten, *J. Mol. Graph.* **1996**, *14*, 33–38.
[17] M. Marianski, A. Supady, T. Ingram, M. Schneider, C. Baldauf, *J. Chem. Theory Comput.* **2016**, *12*, 6157–6168.
[18] N. Troullier, J. L. Martins, *Phys. Rev. B. Condens. Matter* **1991**, *43*, 1993–2006.
[19] A. Ardè, C. Rovira, **2015**, *J. Am. Chem. Soc. 137*, 7528–7547.
[20] G. A. Tribello, M. Bonomi, D. Branduardi, C. Camilloni, G. Bussi, *Comput. Phys. Commun.* **2014**, *185*, 604–613.
[21] B. Ensing, A. Laio, M. Parrinello, M. L. Klein, *J. Phys. Chem. B.* **2005**, *109*, 6676–6687.
[22] I. Nigo Marcos-Alcalde, E. López-Viñas, P. Gómez-Puertas, *Bioinformatics* **2020**, *36*, 956–958.
[23] C. G. Mayne, J. Saam, K. Schulten, E. Tajkhorshid, J. C. Gumbart, *J. Comput. Chem.* **2013**, *34*, 2757–2770.
[24] S. Savino, A. J. E. Borg, A. Dennig, M. Pfeiffer, F. De Giorgi, H. Weber, K. D. Dubey, C. Rovira, A. Mattevi, B. Nidetzky, *Nat. Catal.* **2019**, *2*, 1115–1123.
[25] T. Eixelsberger, B. Nidetzky, *Adv. Synth. Catal.* **2014**, *356*, 3575–3584.
[26] A. J. E. Borg, A. Dennig, H. Weber, B. Nidetzky, *FEBS J.* **2021**, *288*, 1163–1178.
[27] D. Cremer, J. A. Pople, *J. Am. Chem. Soc.* **1975**, *97*, 1354–1358.
[28] X. Gu, S. G. Lee, M. Bar-Peled, *Microbiology* **2011**, *157*, 260–269.
[29] J. B. Thoden, P. A. Frey, H. M. Holden, *Biochemistry* **1996**, *35*, 5137-5144.
[30] J. B. Thoden, H. M. Holden, *Biochemistry* **1998**, *37*, 11469–11477.

[31] V. Carbone, L. R. Schofield, C. Sang, A. J. Sutherland-Smith, R. S. Ronimus, *Proteins Struct. Funct. Bioinforma.* **2018**, *86*, 1306–1312.

[32] N. Ishiyama, C. Creuzenet, J. S. Lam, A. M. Berghuis, *J. Biol. Chem.* **2004**, *279*, 22635–22642.

[33] S. J. Weiner, P. A. Kollman, D. A. Case, U. Chandra Singh, C. Ghio, G. Alagona, S. Profeta, P. Weiner, *J. Am. Chem. Soc.* **1984**, *106*, 765–784.

6. Author Contributions

A.J.E.B. designed the enzyme variants and performed all the experimental work. O.E., J.C. and C.R. performed the MD simulations and QM calculations. B.N. and C.R. supervised the research. B.N., A.J.E.B. and C.R. wrote the paper.
